# One pot synthesis of GDP‐mannose by a multi‐enzyme cascade for enzymatic assembly of lipid‐linked oligosaccharides

**DOI:** 10.1002/bit.26454

**Published:** 2017-10-23

**Authors:** Thomas F.T. Rexer, Anna Schildbach, Jan Klapproth, Angelika Schierhorn, Reza Mahour, Markus Pietzsch, Erdmann Rapp, Udo Reichl

**Affiliations:** ^1^ Max Planck Institute for Dynamics of Complex Technical Systems Bioprocess Engineering Magdeburg Germany; ^2^ Department of Downstream Processing, Institute of Pharmacy Martin Luther University Halle‐Wittenberg Halle (Saale) Germany; ^3^ Institute of Biochemistry and Biotechnology Martin Luther University Halle‐Wittenberg Halle (Saale) Germany; ^4^ Otto‐von‐Guericke University Magdeburg Chair of Bioprocess Engineering Magdeburg Germany

**Keywords:** cell‐free synthesis, enzymatic catalysis, kinetic modeling, in vitro N‐glycoengineering, nucleotide sugar regeneration

## Abstract

Glycosylation of proteins is a key function of the biosynthetic‐secretory pathway in the endoplasmic reticulum (ER) and Golgi apparatus. Glycosylated proteins play a crucial role in cell trafficking and signaling, cell‐cell adhesion, blood‐group antigenicity, and immune response. In addition, the glycosylation of proteins is an important parameter in the optimization of many glycoprotein‐based drugs such as monoclonal antibodies. In vitro glycoengineering of proteins requires glycosyltransferases as well as expensive nucleotide sugars. Here, we present a designed pathway consisting of five enzymes, glucokinase (Glk), phosphomannomutase (ManB), mannose‐1‐phosphate‐guanyltransferase (ManC), inorganic pyrophosphatase (PmPpA), and 1‐domain polyphosphate kinase 2 (1D‐Ppk2) expressed in *E. coli* for the cell‐free production and regeneration of GDP‐mannose from mannose and polyphosphate with catalytic amounts of GDP and ADP. It was shown that GDP‐mannose is produced at various conditions, that is pH 7–8, temperature 25–35°C and co‐factor concentrations of 5–20 mM MgCl_2_. The maximum reaction rate of GDP‐mannose achieved was 2.7 μM/min at 30°C and 10 mM MgCl_2_ producing 566 nmol GDP‐mannose after a reaction time of 240 min. With respect to the initial GDP concentration (0.8 mM) this is equivalent to a yield of 71%. Additionally, the cascade was coupled to purified, transmembrane‐deleted Alg1 (ALG1ΔTM), the first mannosyltransferase in the ER‐associated lipid‐linked oligosaccharide (LLO) assembly. Thereby, in a one‐pot reaction, phytanyl‐PP‐(GlcNAc)_2_‐Man_1_ was produced with efficient nucleotide sugar regeneration for the first time. Phytanyl‐PP‐(GlcNAc)_2_‐Man_1_ can serve as a substrate for the synthesis of LLO for the cell‐free in vitro glycosylation of proteins. A high‐performance anion exchange chromatography method with UV and conductivity detection (HPAEC‐UV/CD) assay was optimized and validated to determine the enzyme kinetics. The established kinetic model enabled the optimization of the GDP‐mannose regenerating cascade and can further be used to study coupling of the GDP‐mannose cascade with glycosyltransferases. Overall, the study envisages a first step towards the development of a platform for the cell‐free production of LLOs as precursors for in vitro glycoengineering of proteins.

**Abbreviations**
1D‐Ppk2: 1‐domain polyphosphate kinase 2
ADP: adenosine diphosphate
Alg1ΔTM: transmembrane‐deleted β‐1,4‐mannosyltransferase
APTS: (3‐Aminopropyl)triethoxysilane
ATP: adenosine triphosphate
CMP‐sialic: cytidine‐5′‐monophospho‐N‐acetylneuraminic acid
DTT: dithiothreitol
ER: endoplasmic reticulum
ESI: electrospray ionization
Glk: glucokinase
GDP: guanosine diphosphate
GDP‐man/GDPM: Guanosine diphosphate mannose
GlcNAc: N‐Acetylglucosamine
GTP: guanosine triphosphate
HCl: hydrochloride
HILIC: hydrophilic interaction chromatography
His‐*tag*: hexa histidine‐tag
HPAEC‐UV/CD: high‐performance anion‐exchange chromatography with ultraviolet and conductivity detection
IgG: immunoglobulin G
IMAC: immobilized metal affinity chromatography
IPTG: isopropyl β‐D‐1‐thiogalactopyranoside
LC: liquid chromatography
LLO: lipid‐linked oligosaccharide
man1P: mannose‐1‐phosphate
man6P: mannose‐6‐phosphate
manB: phosphomannomutase
manC: mannose‐1‐phosphate‐guanyltransferase
MS: mass spectrometry
OD_600_: absorbance at wavelength 600 nm
OST: oligosaccharyl transferase
P: phosphate
phytanyl‐PP‐(GlcNAc)_2_: phytanyl‐pyrophosphate‐chitobiose
phytanyl‐PP‐(GlcNAc)_2_‐Man_1_: phytanyl‐pyrophosphate‐mannosylchitobiose
PmPpA: inorganic pyrophosphatase
PolyP_14_: polyphosphate, average chain length 14 phosphate units
rpm: rounds per min
Q‐TOF: hybrid quadrupole tandem time‐of‐flight
RP: reversed phase
SDS–PAGE: sodium dodecyl sulfate polyacrylamide gel electrophoresis
SPE: solid‐phase extraction
UDP‐galactose: uridindiphosphate‐galactose
UDP‐Glc: uridindiphosphate‐glucose
UDP‐GlcNAc: uridindiphosphate‐N‐Acetylglucosamine
xCGE‐LIF: multiplexed capillary gel electrophoresis with laser‐induced fluorescence

## 1 INTRODUCTION

*N*‐linked protein glycosylation is a co‐translational modification in eukaryotes that affects protein folding directly or indirectly (Culyba et al., 2011; Hanson et al., 2009; Helenius & Aebi, 2004; Shental‐Bechor & Levy, 2008). *N*‐linked glycans play a role in protein stability, solubility and cell trafficking as well as cell signaling (Taylor & Drickamer, 2011). Therefore, the glycosylation of proteins is also an important parameter in the optimization of animal cell culture‐derived drugs including monoclonal antibodies, growth factors, and hormones (Dekkers et al., 2016; Hossler, Khattak, & Li, 2009; Lalonde & Durocher, 2017; Sha, Agarabi, Brorson, Lee, & Yoon, 2016; Spearman, Rodriguez, Huzel, Sunley, & Butler, 2007). In addition, over the past years efforts have been made to modify the *N*‐glycosylation machinery in yeast and *E. coli* for the production of therapeutic proteins at low‐costs with tailored glycosylation in vivo (Srichaisupakit, Ohashi, Misaki, & Fujiyama, 2015; Valderrama‐Rincon et al., 2012; Wildt & Gerngross, 2005). An alternative approach is the in vitro glycoengineering of proteins by modifying the glycostructure via enzymatic reactions with purified glycosyltransferases and nucleotide sugars (Thomann et al., 2015). Case studies have shown very promising results in terms of increasing the level of galactosylation and sialylation on IgG (Chung et al., 2006; Raju, Briggs, Chamow, Winkler, & Jones, 2001; Thomann et al., 2015). To satisfy the high demand of nucleotide sugars UDP‐galactose and CMP‐sialic acid for in vitro glycoengineering, Raju et al. (2001) have designed an in vitro enzymatic nucleotide sugar regeneration cascade for these two co‐substrates and demonstrated galactosylation and sialylation of tumor necrosis factor receptor IgGs.

In order to in vitro *N*‐glycosylate proteins by enzymatic reactions, lipid‐linked oligosaccharides (LLO) are needed as substrates (Ramírez, Boilevin, Biswas, et al., 2017; Ramírez, Boilevin, Lin, et al., 2017). The ER associated biosynthesis of the LLO is a highly conserved process in eukaryotic cells. The core glycan (GlcNAc)_2_‐Man_9_‐Glc_3_ is assembled on a membrane‐localized dolichyl‐pyrophosphate by a cascade of 12 glycosyltransferases and is then transferred to a nascent polypeptide chain by an oligosaccharyltransferase (OST). GDP‐mannose (GDP‐man), UDP‐GlcNAc and UDP‐Glc serve, directly and indirectly, as mannose, N‐acetylglucosamine and glucose donors for the attachment of sugars to the LLO (Aebi, 2013; Helenius & Aebi, 2004). So far, to the best of our knowledge, there is no cell‐free platform or process for the preparative synthesis of ER‐LLOs with efficient nucleotide sugar regeneration. Challenges are, in particular, the expression and the purification of ER membrane‐associated glycosyltransferases, and the provision of key enzymatic reactions with expensive sugar nucleotides, namely GDP‐man, UDP‐GlcNAc, and UDP‐Glc. GDP‐man is enzymatically produced from mannose‐1‐phosphate and GTP by mannose‐1‐phosphate guanyltransferase (ManC). In nature there are two pathways for the production of mannose‐1‐phosphate starting either from glucose or mannose in the salvage pathway (Kuettel et al., 2012; Pfeiffer, Bulfon, Weber, & Nidetzky, 2016). Several studies have been published on exploiting and modifying these pathways for the cell‐free production and isolation of GDP‐man (Jia et al., 2011; Pfeiffer et al., 2016; Wang, Shen, Wang, Ichikawa, & Wong, 1993). Honghong et al. have designed an enzyme cascade based on the salvage pathway. Using a raw extract of *E. coli* containing recombinant glucokinase (Glk), phosphomannomutase (ManB), and mannose‐1‐phosphate‐guanyltransferase (ManC) the GDP‐man was produced from mannose, ATP, and GTP (Jia et al., 2011). To avoid product purification after one‐pot multi‐enzyme cascade synthesis of nucleotide sugars, the in vitro coupling of an enzyme cascade regenerating nucleotide sugars to the glycosyltransferase reactions is advantageous. For example, Chung et al. (2006) published 2006 the in vitro galactosylation and sialylation of human IgG in combination with the regenerating enzymes, all expressed in *E. coli* and used as raw extracts. Wang et al. (1993) have designed an in vitro enzyme cascade with pyruvate kinase, inorganic pyrophosphatase, and mannose‐1‐phosphate‐guanyltransferase to regenerate GDP‐man from mannose‐1‐phosphate. The cascade was coupled to α‐1,2‐mannosyltransferase to attach mannose on O‐mannosylglycopeptides.

Here we present a systematic, model‐supported development of a cell‐free synthetic enzyme cascade consisting of five enzymes to synthesize and continuously regenerate GDP‐man from mannose and polyphosphate with catalytic amounts of GDP and ADP. The cascade was optimized for effective GDP‐man production and tested at pH values 7–8, temperatures 25–35°C, and co‐factor concentrations 0–20 mM MgCl_2_ to characterize the synthesis reactions at various conditions. In addition, the cascade was in vitro coupled to a transmembrane‐deleted β‐1,4‐mannosyltransferase (Alg1ΔTM) in a one‐pot reaction to produce phytanyl‐PP‐(GlcNAc)_2_‐Man_1_. To identify inhibition and bottlenecks in the multi‐enzyme cascade reaction a kinetic model was established using the MATLAB® systems biology toolbox.

## 2 MATERIALS AND METHODS

For a comprehensive list of chemicals used including vendors and purity grades, see the supplementary information.

### 2.1 Pathway design

**Figure 1:**
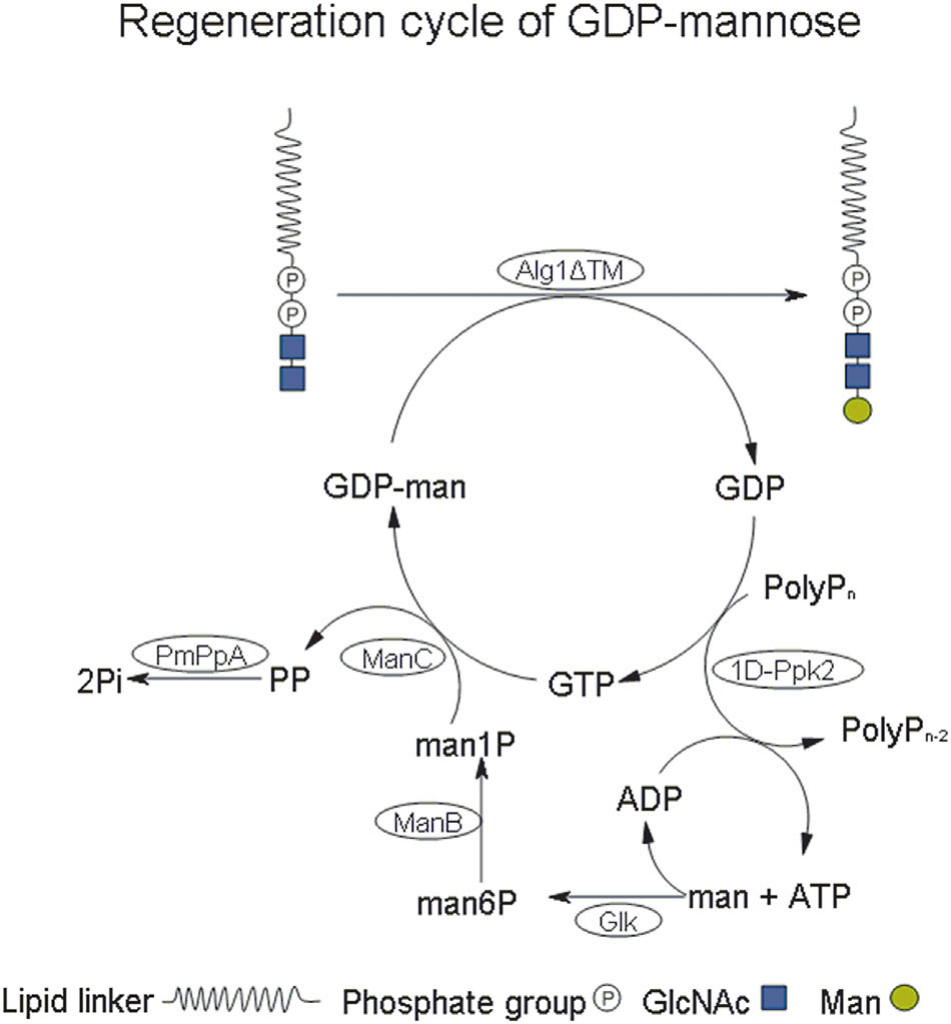
Engineered in vitro GDP‐mannose pathway with enzymes from different microorganisms expressed in *E. coli*: Glucokinase (His_6_‐Glk), phosphomannomutase (ManB‐His_6_) and mannose‐1‐phosphateguanyltransferase (ManC), inorganic pyrophosphatase (PmPpA‐His_6_), 1‐domain polyphosphate kinase 2 (His_6_‐1D‐Ppk2) in vitro coupled to β‐1,4‐mannosyltransferase (Alg1ΔTM) for the production of phytanyl‐PP‐(GlcNAc)_2_‐Man_1_ with GDP‐mannose regeneration

The GDP‐man pathway used (see Figure 1) consists of five enzymes which were separately expressed in and purified from *E. coli* BL21‐Gold (DE3): Glucokinase (Glk) (EC 2.7.1.2), phosphomannomutase (ManB) (EC 5.4.2.8), and mannose‐1‐phosphate‐guanyltransferase (ManC) (EC 2.7.7.13) originating from *E. coli* W3110 (DSM 5911), inorganic pyrophosphatase (PmPpA) (EC 3.6.1.1) originating from *Pasteurella multocida,* and 1‐domain‐polyphosphate kinase 2 (1D‐Ppk2) (EC 2.7.4.1) originating from *Pseudomonas aeruginosa*. Origins of the enzymes were choosen according to the literature (Chen et al., 2011; Koizumi et al., 2000; Lau et al., 2010; Meyer, Schneider‐Fresenius, Horlacher, Peist, & Boos, 1997; Nocek et al., 2008).

### 2.2 Enzyme expression and purification

#### 2.2.1 Strains, plasmids, and media

*E. coli* W3110 (DSM5911, DSMZ, Braunschweig, Germany) was used for amplification of desired genes. *E. coli* BL21‐Gold (DE3) was purchased from Stratagene (Amsterdam, the Netherlands). The plasmid pET‐28a (+) was purchased from Novagen (Darmstadt, Germany).

#### 2.2.2 Cloning of glk and manBC from *E. coli* W3110 into the expression vector pET‐28a (+)

The gene sequence *glk* (GenBank accession number U22490) was amplified from genomic DNA of *E. coli* W3110 using the following primer pair: glk‐for 5′‐GGAATTCCATATGACAAAGTATGCATTAGTCGGTG‐3′ including a *Nde*I restriction site (underlined), glk‐rev 5′‐CCTCGAGCGG‐ACGCAGGTCGACCTTGT‐3′ carrying a *Xho*I restriction site (Meyer et al., 1997). The gene sequences of *manB* (GenBank accession number M77127) and *manC* (GenBank accession number U38473) were amplified according to Koizumi et al. (2000) using the primers manCB‐for 5′‐CATGCCATGGCGCAGTCGAAACTCTATCC‐3′ with *Nco*I restriction site and manCB‐rev 5′‐CCTCGAGCGG‐CTCGTTCAGCAACGTCAG‐3′ with *Xho*I (Primer purchased from Eurofins Genomics (Ebersberg, Germany). Restriction digest, ligation and transformation were performed following standard operating procedures (Sambrook, Fritsch, & Maniatis, 1989).

#### 2.2.3 Gene synthesis of *ppa*, *ppk2* (PA2428), and *ALG1* (Δ4‐105)

The nucleotide and protein sequences of genes *ppa*, *ppk2*, and *ALG1* (Δ4‐105) coding for PmPpA from *Pasteurella multocida*, 1D‐Ppk2 from *Pseudomonas aeruginosa*, and Alg1 from *Saccharomyces cerevisiae* were downloaded from the GenBank database (accession numbers AAK03275, NP_251118, and J05416, respectively). In case of *ALG1* (Δ4‐105) the bases 4–105, coding for the transmembrane domain, were deleted according to Revers, Wilson, Webberley, and Flitsch (1994). All nucleotide sequences were codon usage optimized (Software: Gene Designer, DNA 2.0, Menlo Park, CA) for expression in *E. coli*. Restriction sites for subcloning into pET‐28a (+) were added to the sequences as follows: *ppa* (*Nco*I, *Xho*I), *ppk2* (*Nde*I, *Sac*I), and *ALG1* (Δ4‐105) (*Nde*I, *Xho*I) (Villalobos, Ness, Gustafsson, Minshull, & Govindarajan, 2006). The resulting sequences were synthesized de novo and cloned into pET‐28a (+) by GeneArt from Thermo Fisher Scientific (Regensburg, Germany). Prior to transformation of the received plasmids into *E. coli* BL21‐Gold (DE3) the accuracy of the constructs was checked by sequencing (Eurofins Genomics, Ebersberg, Germany—see supplementary information for gene and protein sequences).

#### 2.2.4 Cultivation of enzyme variants in *E. coli* BL21‐Gold (DE3)

Transformants were grown in 1 L shaking flasks with baffles in a volume of 500 ml of LB medium supplemented with 50 µg/ml Kanamycin. The cultures were grown at 37°C (His_6_‐Glk, ManB‐His_6_‐ManC, His_6_‐Alg1ΔTM, PmPpA‐His_6_) and 24°C (His_6_‐1D‐Ppk2), respectively, and shaken at 80 rpm. The induction of the LacZ promotor was forced by addition of IPTG with a final concentration of 1 mM to the culture at an OD_600_ of 0.5–0.6. Expression time was terminated after 4 hr. Biomass was separated from the medium by centrifugation at 6,000 × *g* for 10 min. Successful expression of the respective protein was analyzed by SDS–PAGE following standard operating procedures (Laemmli, 1970). The wet biomass was stored at −20°C.

#### 2.2.5 Purification of enzymes by immobilized metal affinity chromatography

For purification, typically 30 ml of equilibration buffer were added to 3 g of frozen biomass. The equilibration buffer consisted of 50 mM Tris/HCl (pH 7.5), 500 mM NaCl, 10 mM imidazole, and 10 mM MgCl_2_. In case of His_6_‐1D‐Ppk2 purification, 5 % glycerol (v/v) was added to stabilize the enzyme (Bradbury & Jakoby, 1972) according to (Zhang et al., 2001). For purification of His_6_‐Alg1ΔTM, the concentration of imidazole in the equilibration buffer was 30 mM; in addition the buffer contained 0.25% (w/v) of Triton X–100. Following thawing at 4°C under stirring, cells were disrupted by four passages through a high pressure homogenizer (Emulsiflex C5, Avestin Inc., Ottawa, Canada) at 1,000 bar with intermediate cooling on ice. After centrifugation (45 min, 20,000 × g), the supernatant was applied to an equilibrated Immobilized Metal Affinity Chromatography (IMAC) column (10 ml CV) containing Ni^2+^ Sepharose™ High Performance chromatography material from Amersham Biosciences (Uppsala, Sweden). Unbound proteins were washed out using equilibration buffer. Immobilized protein was eluted in 1 ml fractions using elution buffer containing 50 mM Tris/HCl (pH 7.5), 500 mM NaCl, 500 mM imidazole, and 10 mM MgCl_2_ with changes in equilibration buffer composition also applied to the elution buffer. To remove excess imidazole, the eluted pool (typically 10 ml) was dialyzed two times against 3 L of reaction buffer containing 20 mM Tris/HCl (pH 7.5), 50 mM NaCl, and 10 mM MgCl_2_ (again with respect to the above mentioned changes). Finally, the enzyme solutions were concentrated by Centrifugal Filter Units Amicon**®** Ultra‐15 with a 50 kDa cut‐off from Merck Millipore (Darmstadt, Germany). No enzyme loss was observed during the ultrafiltration. The enzymes were stored in 50% glycerol at −20°C. The protein concentration was determined by Bradford assay using BSA as standard (Bradford, 1976).

### 2.3 Analytics

#### 2.3.1 Protein analysis

Protein bands were analyzed by in‐gel trypsin digestion and Nano‐HPLC‐ESI‐MS/MS analysis. SYNAPT® G2 MS, a Quadrupole quadrupole time‐of‐flight (Qq‐Tof) hybrid mass spectrometer (MS), from Waters Co. (Milford) was used for the MS/MS analysis.

#### 2.3.2 Chromatography

**Figure 2:**
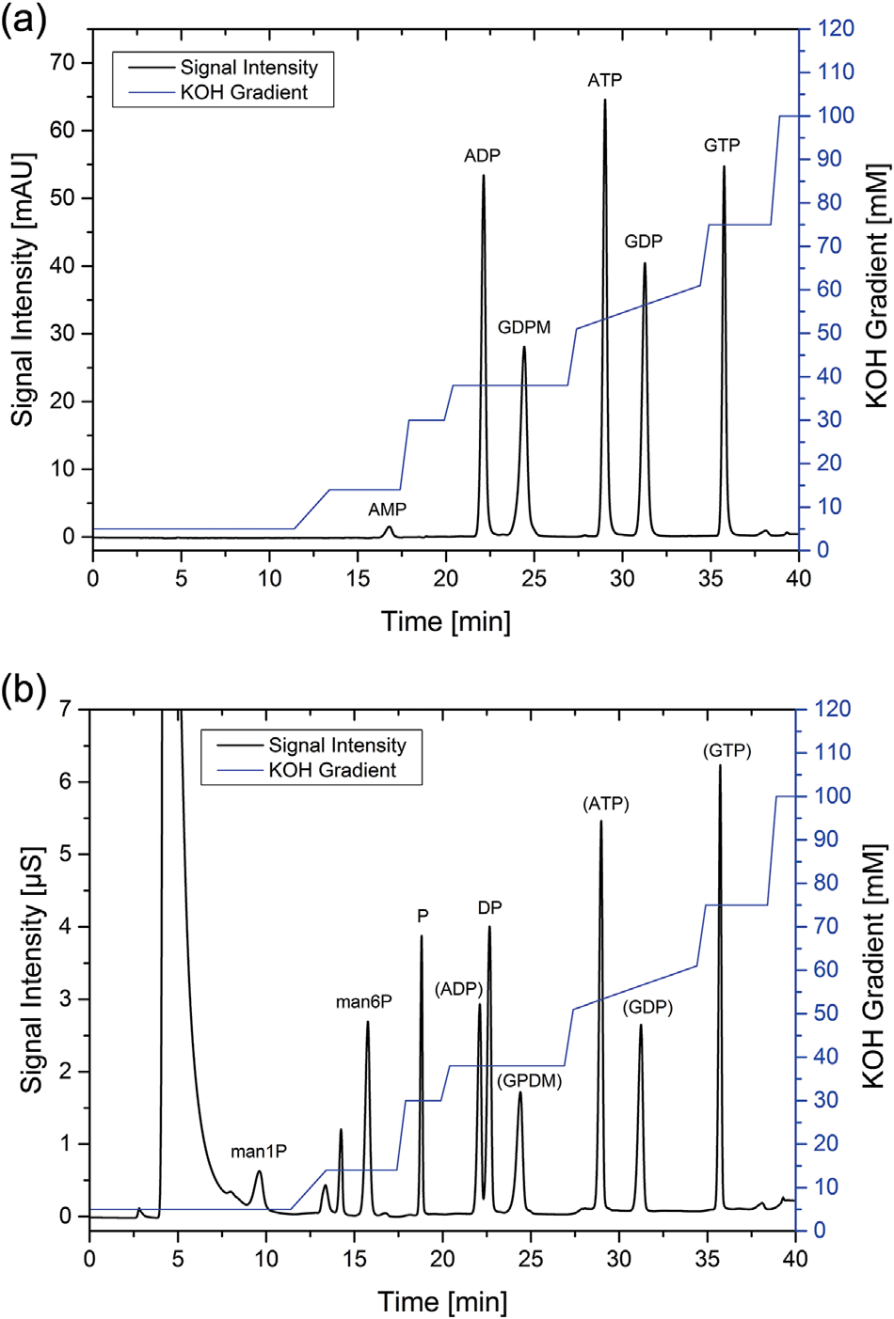
(a) UV‐Chromatogram of a 170 µM standard sample for the developed and optimized elution (KOH) gradient. The UV wavelength for detection was 260 nm. ADP, GDP‐mannose, ATP, GDP, and GTP concentrations were quantified by UV signals. The assay was validated. (b) Conductivity chromatogram of a 170 µM standard sample for the developed and optimized elution (KOH) gradient. Suppression of hydroxide ions was conducted by the electronically regenerated suppressor ERS500 from Dionex. The large peak at 5 min was caused by Cl^−^ ions. Man1P, man6P, phosphate (P), and diphosphate (DP) concentrations were measured by conductivity detection. The assay was validated

Reaction substrates and products were separated and quantified by high performance anion‐exchange chromatography (HPAEC). A BioLCType DX320 system from Dionex (Sunnyvale) with UV (wave length: 260 nm) and conductivity detection was used. Chromatographic separation was performed at a system flow of 0.35 ml/min by two analytical columns, AS11 (250 × 2 mm) operated in‐series. The eluent gradient (5–100 mM KOH) for chromatographic separation was based on previous studies with some modifications (Ritter, Genzel, & Reichl, 2006). The gradient is shown in Figures 2a and 2b. All columns, components and software were purchased from Thermo Scientific (Waltham).

ADP, ATP, GDP, GTP, and GDP‐man concentrations were measured by UV‐detection (Figure 2a). Mannose‐1‐phosphate (man1P), mannose‐6‐phosphate (man6P), phosphate, and pyrophosphate were measured by conductivity detection (Figure 2b). For assay validation and more details on chromatography see the attached supplement.

#### 2.3.3 Reaction conditions

Reaction volumes for kinetic measurements were 1 ml. All reactions were carried out with a co‐factor concentration of 10 mM MgCl_2_ at 30°C in 50 mM Tris/HCl buffer of pH 7.5 and incubated at 30 rpm in a thermomixer from Eppendorf AG (Hamburg, Germany) unless stated otherwise. Sample aliquots of 100 μl were quenched in 400–900 μl of MilliQ water, preheated in a closed Eppendorf tube to 90°C, followed by another 10 min of heating at 90°C. To ensure enzyme inactivation, the quenching protocol was tested on all enzymes.

All reactions were tested for reversibility, inhibition, and long‐term stability when stored as well as enzyme inactivation during assaying by Selwyn's Test (Selwyn, 1965). Overall six data sets—Glk (16 reactions), PmPpa (13 reactions), 1d‐Ppk2 (25 reactions), ManB/C (42 reactions), ALG1ΔTM (8 reactions), multi‐enzyme cascade (16 reactions)—were generated and are available on request.

#### 2.3.4 Glycan analysis

To detect (GlcNAc)_2_‐Man_1_ multiplexed capillary gel electrophoresis with laser‐induced fluorescence detection (xCGE‐LIF) was utilized (Hennig et al., 2015; Rapp, Hennig, Borowiak, Kottler, & Reichl, 2011; Ruhaak et al., 2010; Schwarzer, Rapp, & Reichl, 2008). Mild acid hydrolysis was carried out by mixing the vacuum‐dried and in 150 μl isopropanol resuspended sample aliquots (100 μl) with 150 μl HCl (40 mM). The mix was incubated for 45 min at 95°C before neutralization with 50 μl NaOH (100 mM) followed. Standard protocols were followed to fluorescently label samples with APTS and to subsequently remove excess APTS by hydrophilic interaction chromatography with solid phase extraction (HILIC‐SPE) (Hennig et al., 2015). 2 μl aliquots were mixed with 2 μl HiDi™, 2 μl LIZ™ base pair standard, and 2 μl 2nd NormMix and injected for 5 s with 15 kV and 30°C on a 4‐capillary DNA‐sequencer ABI PRISM 3100‐Avant Genetic Analyzer with a POP‐7™ (50 cm) polymer matrix, both from Applied Biosystems (Waltham, MA). The commercial software glyXtool™ from GlyXera™ (Magdeburg, Germany) was used for data normalization and analysis. The LIZ™ base pair standard is used for a first migration time normalization and the 2nd NormMix is utilized to refine migration time normalization according to literature (Hennig et al., 2016).

### 2.4 Data‐fitting and simulations

The systems biology toolbox SBTOOLBOX2 for MATLAB® (Version R2013b) from The MathWorks (Natick) was used for data fitting and simulations (Schmidt, 2007; Schmidt & Jirstrand, 2006). Typically, the adjusted Nelder‐Mead Simplex and the Particle swarm pattern search algorithm were employed interchangeably to find function minima (Olsson & Nelson, 1975; Press, Teukolsky, Vetterling, & Flannery, 1996; Vaz & Vicente, 2007). For data fitting one fit per data set was performed.

## 3 RESULTS AND DISCUSSION

### 3.1 Enzyme expression and purification

Using the indicated restriction sites for ligation into pET‐28a (+), Glk was expressed in *E. coli* BL21‐Gold (DE3) with a 6‐fold N‐terminal (His_6_‐Glk), ManB with a C‐terminal Histidin‐*tag* (ManB‐His_6_) and ManC without affinity *tag*. The enzymes His_6_‐1D‐Ppk2 and His_6_‐Alg1ΔTM carried an *N*‐terminal sixfold His‐*tag* (His_6_‐1D‐Ppk2, His_6_‐Alg1ΔTM) and PmPpA a C‐terminal His‐*tag* (PmPpA‐His_6_). All enzymes were produced as soluble proteins.

His_6_‐Glk was produced as mentioned in the section 2 (Lunin et al., 2004; Meyer et al., 1997). From 2 g bio wet mass, 164 mg highly pure enzymes were isolated. The yield was 27‐fold higher compared to the literature (Lunin et al., 2004). Aqueous enzyme stock solution contained 8.2 mg/ml His_6_‐Glk.

PmPpA‐His_6_ was produced according to the literature (Lau et al., 2010). The enzyme precipitated during the dialysis and was therefore used in non‐dialyzed form. From 2 g bio wet mass, 53 mg highly pure enzyme was isolated. PmPpA‐His_6_ concentration in the stock solution was 2.03 mg/ml.

ManC and ManB‐His_6_ were co‐expressed according to the literature using one plasmid, separated by a 50 bp linker region, each nucleotide sequence having its own ribosomal binding site (Koizumi et al., 2000; Lee, Han, Park, & Seo, 2009). So far, both enzymes were applied as crude enzymes or as raw extracts (Koizumi et al., 2000; Lee et al., 2009). In order to prevent side‐reactions from other host proteins, a purification protocol was developed based on IMAC. Untagged ManC was co‐eluted with the adsorbed ManB‐His_6_. This result can be either explained by an intermolecular interaction of both enzymes or by the 14 histidines present in the amino acid sequence of ManC (see supplement). Eight of these histidine residues are located in the C‐terminal part in close vicinity to each other. The enzymes were not separated before use and were free of contaminating host cell proteins from *E. coli*. From 3 g bio wet mass, 74 mg highly pure enzymes were isolated. Enzyme stock solution contained 1.71 mg/ml ManB‐His6/ManC.

His_6_‐1D‐Ppk2 was produced according to the literature (Nocek et al., 2008; Zhang et al., 2001). During expression of His_6_‐1D‐Ppk2 at 37°C the formation of inclusion bodies was detected. Lowering the temperature to 24°C after induction increased the yield of the soluble enzyme substantially. After purification, His_6_‐1D‐Ppk2 was soluble in concentrations of about 0.5 mg/ml in presence of 50% glycerol. Above this concentration the enzyme precipitated. From 3 g bio wet mass, 25 mg highly pure enzyme was isolated. His_6_‐1D‐Ppk2 stock solution contained 0.15 mg/ml of the enzyme.

His_6_‐Alg1ΔTM was produced according to the literature using 0.25 % Triton X–100 as a stabilizer (Revers, Bill, Wilson, Watt, & Flitsch, 1999). Without the addition of Triton X–100 to the binding buffer His_6_‐Alg1ΔTM was not bound to the IMAC stationary phase. The addition of 5 mM DTT to the dialysis buffer solved the problem of inactivation/precipitation. From 3 g bio wet mass, 5 mg highly pure enzyme was isolated and the stock solution contained 0.55 mg/ml His_6_‐Alg1ΔTM after purification and addition of glycerol.

**Figure 3:**
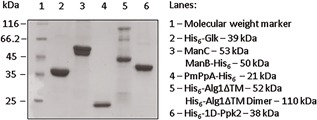
Analysis of the degree of purity of enzymes used for the GDP‐mannose regeneration cycle (SDS–PAGE, Coomassie stained). The concentrations of the purified proteins were 0.4–0.7 mg/ml. Lane 1–Molecular weight marker (PageRulerTM Unstained Low Range Protein Ladder, Thermo Fisher Scientific, Schwerte)

All purified enzymes were analyzed by SDS–PAGE (Figure 3). The predicted molecular weights of the target proteins are as follows: His_6_‐Glk: 39 kDa, ManB‐His_6_: 50 kDa, ManC: 53 kDa, His_6_‐1D‐Ppk2: 38 kDa, PmPpA‐ His_6_: 21 kDa, and His_6_‐Alg1ΔTM: 52 kDa. Except for His_6_‐Alg1ΔTM, all enzymes produced were purified to homogeneity. The purity of His_6_‐Alg1ΔTM was lower than the purity of the other five enzymes. Despite the deletion of the transmembrane domain, His_6_‐Alg1ΔTM exhibits high hydrophobicity. Binding to the IMAC column material could only be facilitated in the presence of detergent Triton X–100 in concentrations higher than the critical micelle concentration (0.22–0.24 mM). Accordingly, other proteins from *E. coli* bound to Triton X–100 micelles are present in His_6_‐Alg1ΔTM stock solutions. Due to the high hydrophobicity His_6_‐Alg1ΔTM shows unusual migration behavior in SDS–PAGE. The calculated molecular weight of His_6_‐Alg1ΔTM is 52.6 kDa, but the protein band is detected at 45 kDa. The 45 kDa band and the band that was detected at around 100 kDa in the same lane were analyzed by LC‐ESI‐MS after tryptic digestion (see supplementary information for sequence coverage). Both bands belong to His_6_‐Alg1ΔTM, the 100 kDa band probably represents a dimer of the protein. The yield of purified enzymes was up to 246 mg of His_6_‐Glk, 80 mg of a mixture of ManC and ManB‐His_6_, 18 mg of His_6_‐Alg1ΔTM, 15 mg of His_6_‐1D‐Ppk2, and 86 mg of PmPpA‐His_6_ per 1 L of cell culture prepared in shaking flasks.

### 3.2 Single‐enzyme kinetics

The kinetics of enzyme‐catalyzed in vitro reactions are influenced by multiple parameters such as enzyme concentration, purity, and buffer components. Published reaction conditions such as temperature, pH value, co‐factors, co‐factor concentration, and type of reaction buffers for different enzymes differ significantly. Therefore, in the present study, the enzyme kinetics of each reaction was determined experimentally for the reaction conditions stated in the section 2.

**Figure 4:**
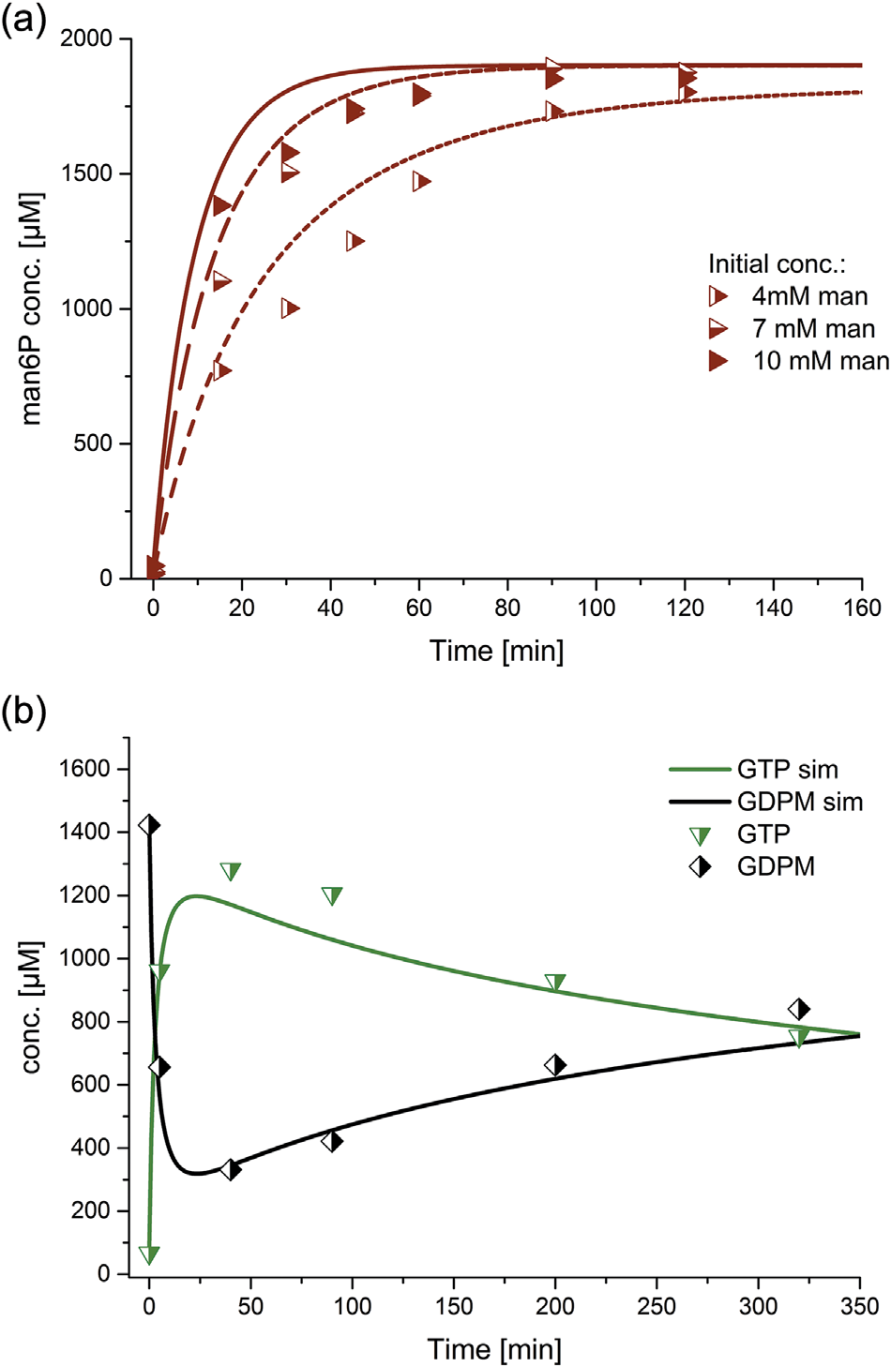
(a) Irreversible man6P synthesis by His_6_‐Glk (1 mg/ml) from ATP (1.7 mM) using different mannose concentrations. The lines represent the model (see Table 1). (b) Reverse reaction of the ManB‐His_6_/ManC (0.171 mg/ml) complex: GDP‐mannose and pyrophosphate are converted to GTP and man6P catalyzed by ManB and ManC. Subsequently, the synthesis of the GDP‐man is largely dependent on the conversion of pyrophosphate to phosphate. The fits depicted are fits to the entire data set of His_6_‐Glk and ManB‐His_6_/ManC, respectively, catalyzed reactions

**Figure 5:**
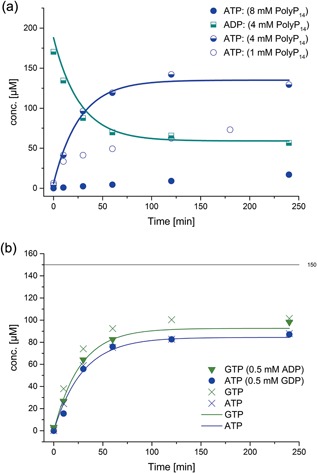
(a) Phosphorylation of ADP and GDP by His_6_‐1D‐Ppk2 (0.05 mg/ml): ATP concentration profile (ADP, GDP, and GTP not shown) with various initial PolyP_14_ concentrations. Starting concentrations were 0.2 mM ADP, 0.2 mM GDP, and various PolyP_14_ conc. (see legend). The lines represent the model based on mass action kinetics. It was not possible to obtain a simple kinetic rate law to model the reaction inhibition of high and low concentrations of PolyP_14_. (b) The phosphorylation reactions of ADP and GDP are independent of each other at the investigated conditions. Starting concentrations: Blue cross: 0.15 mM ADP versus blue circles: 0.15 mM ADP, 0.5 mM GDP; green cross: 0.15 mM GDP versus green triangles: 0.15 mM GDP, 0.5 mM ADP. The lines represent the concentrations of GTP (green) and ATP (blue) obtained from simulations. 150 µM is the highest possible GTP and ATP conc., respectively, according to the mass balance. The fits depicted are fits to the entire data set of His_6_‐1D‐Ppk2 catalyzed reactions

**Figure 6:**
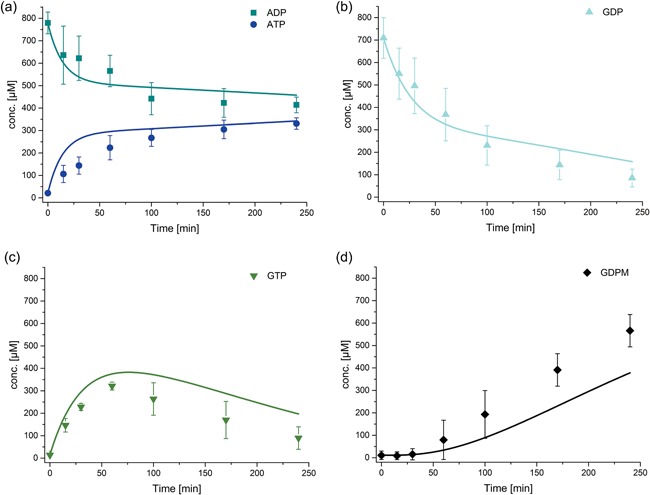
Cascade reaction for the production of GDP‐mannose starting from mannose, ADP, GDP, and PolyP_14_–experimental (dots) and simulated data (lines). Enzyme concentrations are His_6_‐Glk 0.5 mg/ml, His_6_‐1DPpk2 0.05 mg/ml, ManB‐His_6_/ManC 0.3 mg/ml, and PmPpA‐His_6_ 0.1 mg/ml and initial concentrations ADP 0.8 mM, GDP 0.8 mM, mannose 6 mM, and PolyP_14_ 4 mM. Reaction conditions: pH 7.5, 30°C and 10 mM MgCl_2_. The error bars represent the standard deviation of the mean calculated from four replicates. The fits depicted are fits to the entire data set of ManB‐His_6_/ManC catalyzed reactions. (a) ADP and ATP concentrations, (b) GDP concentration, (c) GTP concentration, (d) GDP‐man concentration

**Figure 7:**
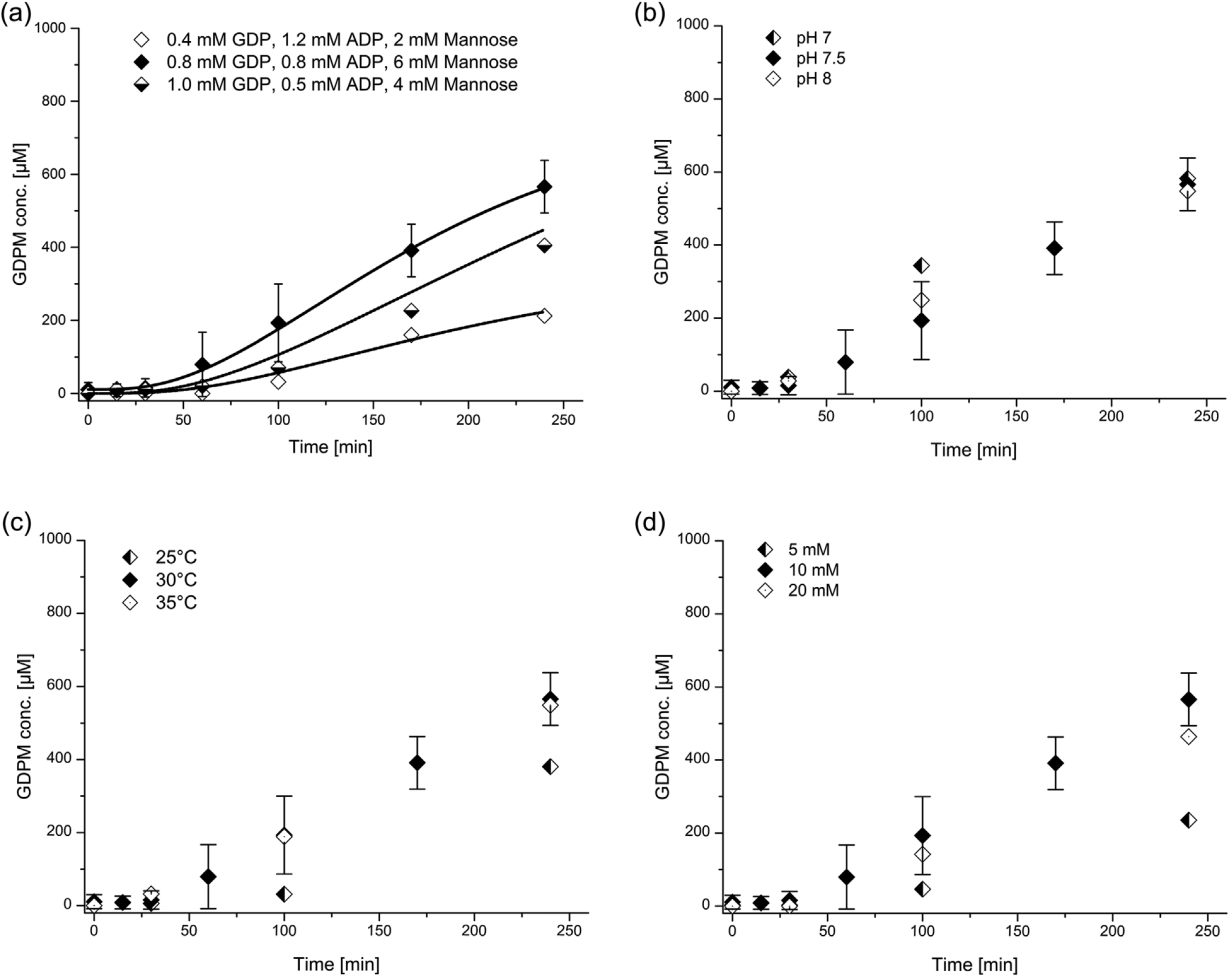
GDP‐mannose concentration over time for cascade reaction starting from mannose, ADP, GDP, and PolyP_14_. Enzyme concentrations were His_6_‐Glk 0.5 mg/ml, His_6_‐1D‐Ppk2 0.05 mg/ml, ManB‐His6/ManC 0.3 mg/ml, and PmPpA‐His_6_ 0.1 mg/ml. Initial substrate concentrations are ADP 0.8 mM, GDP 0.8 mM, mannose 6 mM, and PolyP_14_ 4 mM; reaction conditions were pH 7.5, 30°C and 10 mM MgCl_2_ unless stated otherwise. The error bars of the standard reaction represent the standard deviation of the mean calculated from four replicates. (a) Experimental data (dots) and modeled data with k_4,opt_ (lines) for various initial concentrations; (b) GDP‐mannose concentration for reactions at pH values 7.0, 7.5, and 8.0; (c) GDPmannose concentration for reactions at 25, 30, and 35°C; (d) GDP‐mannose concentrations for MgCl_2_ concentrations of 5, 10, and 20 mM. The fits depicted are fits to the entire data set of multi‐enzyme catalyzed reactions

The kinetic parameters for each enzyme were determined by fitting the generated experimental data sets to the equations detailed in Table 1. Due to the large amount of experimental data only a selection of reactions and fits are shown here (see Figures 4–7 and 9). All experimental kinetic data is available on request. In the following, the results for the single enzymes are discussed.

**Table 1:** Simple models based on mass action kinetics to describe the experimental data

Enzyme	EC No.	Catalyzed reaction	Reaction rate equation	Estimated parameters	
His_6_‐Glk	2.7.1.2	man + ATP→ man6P + ADP	$r_{1}=k_{1}\;e_{glk}\left[man\right]\left[ATP\right]$	k_1 _= 11.320	L^2^/(min g mol)
His_6_‐1D‐Ppk2	2.7.4.1	PolyP_14 _+ ADP↔ PolyP_13 _+ ATP	$r_{2}=k_{2}\;e_{pk2}\left([GDP]-\frac{\left[GTP\right]}{K_{eq1}}\right)$	k_2 _= 0.519 K_eq1_ = 2.289 k_3 _= 0.439 K_eq2 _= 1.816	L/(min g) L/(min g)
		PolyP_14 _+ GDP↔PolyP_13 _+ GTP	$r_{3}=k_{3}\;e_{pk2}\left([ADP]-\frac{\left[ATP\right]}{K_{eq2}}\right)$		
ManB‐His_6_/ManC	5.4.2.8 2.7.7.13	GTP + man6P ↔GDP‐man + PP PP + H_2_O→ 2 Pi	$r_{4}=k_{4}\;e_{man}\left([man6P][GTP]-\frac{[PP]\left[GDPM\right]}{k_{eq3}}\right)$ $r_{5}=k_{5}{\;\;e}_{man}\left[DP\right]$	k_4 _= 18.163 K_eq3 _= 0.032 k_5 _= 0.130	L^2^/(min g mol) L/(min g)
PmPpA‐His_6_	3.6.1.1	PP + H_2_O→ 2 Pi	*r*_6 _= instant conversion	k_6_ = 13.152	L/(min g)
			$r_{7}=k_{6}\;e_{man}\left[DP\right]$		
His_6_‐Glk, His_6_‐1D‐Ppk2, ManB‐His_6_/ManC, PmPpA‐His_6_		PolyP_14 _+ man + GDP→ GDP‐man + PolyP_12_	$r_{8}=r_{1}+r_{2}+r_{3}+r_{4}+r_{5}+r_{6}+r_{7}$	k_4,opt _= 0.005	L^2^/(min g mol)
ALG1ΔTM		phyt‐PP‐(GlcNAc)_2 _+ GDP‐man→ phyt‐PP‐(GlcNAc)_2_‐man_1 _+ GDP	$r_{ALG1}=k_{7}\;e_{ALG1}{\left[{GDPM}^{}\right]}^{2}{\left[Phyt{\left(GlcNAc\right)}_{2}\right]}^{2}$	k_7 _= 8.1 · 10^9^	L^4^/(min g mol^3^)
His_6_‐Glk, His_6_‐1D‐Ppk2, ManB‐His_6_/ManC, PmPpA‐His_6_, ALG1ΔTM		phyt‐PP‐(GlcNAc)_2 _+ PolyP_14 _+ man + GDP→phyt‐PP‐(GlcNAc)_2_‐man_1 _+ PolyP_12_	$r_{9}=r_{8}+r_{ALG1}$	k_2,opt _= 0.097 k_3.opt_ = 0.041	L/(min g) L/(min g)

The parameters were estimated by fitting the experimental data sets of single and multi‐enzyme reactions, respectively, to the equations. Due to the large amount of data not all reactions and fits can be depicted. Denotation: *r*_i_ reaction rate, *k*_i_ rate constant for mass action kinetics, *e*_i_ enzyme concentration, [i] metabolite concentration, and *K*_eqi_ equilibrium constant. PP = pyrophosphate.

#### 3.2.1 His_6_‐Glk

Glk catalyzes the reaction of glucose and ATP to glucose‐6‐phosphate and ADP. Previous studies of His_6_‐Glk from *E. coli* primarily focused on the conversion of glucose. Instead of glucose, the enzyme also accepts mannose as a substrate. However, experimental data on the enzyme kinetics are very scarce. The only report states that the maximum rate of mannose conversion is “reduced” compared to glucose. For mannose conversion kinetic data are missing (Arora & Pedersen, 1995; Lunin et al., 2004; Meyer et al., 1997; Miller & Raines, 2004).

In our experiments, it was found that the His_6_‐Glk‐catalyzed conversion of ATP and mannose to man6P and ADP is irreversible. Reactions with His_6_‐Glk concentrations of 1 mg/ml (25.6 µM) were not inhibited by mannose (up to 10 mM) (Figure 4A). There was no loss of enzyme activity over 11 months when stored at −20°C. No man6P was produced when His_6_‐Glk (1 mg/ml) was incubated with 2 mM of GTP. Reactions with His_6_‐Glk concentrations of 0.5–2 mg/ml (ATP: 2 mM, mannose: 4 mM) over a time course of up to 180 min revealed no enzyme inactivation according to Selwyn's plot (data not shown). The experimental data were fitted to mass action kinetics and a good fit was achieved.

#### 3.2.2 PmPpA‐His_6_

PmPpA from *P. multocida* catalyzes the hydrolysis of pyrophosphate and has not been characterized and kinetically investigated yet. Own pre‐tests at 37°C measured by UV spectroscopy with pyrophosphate concentrations between 0.015–0.225 mM confirmed the activity of the PmPpA‐His_6_ (see supplementary information). At the employed concentrations of PmPpA‐His_6_ (0.445 μg/ml; equivalent to 21.2 nM) the reaction rate to mass ratio is several orders of magnitude higher compared to the other enzymes of the cascade. In the model, it was therefore assumed that pyrophosphate is consumed instantaneously. For more details on the kinetics see the supplementary information.

#### 3.2.3 His_6_‐1D‐Ppk2

His_6_‐1D‐Ppk2 from *P. aeruginosa* catalyzes the synthesis of both ATP and GTP from ADP and GDP, respectively, as one substrate with inorganic polyphosphate as the second substrate. The enzyme is dependent on Mg^2+^ ions and was characterized and kinetically investigated first by (Ishige, Zhang, & Kornberg, 2002; Zhang, Ishige, & Kornberg, 2002). Later it was discriminated from the two‐domain Ppk2 (2D‐Ppk2) which catalyzes the phosphorylation of AMP and GMP (Nocek et al., 2008).

In our experiments it was found that the phosphorylation of ADP and GDP, respectively, by His_6_‐1D‐Ppk2 are equilibrium reactions (see Figure 5). This is in disagreement with Nocek et al. (2008) who did not observe reversibility. Quantification of the polyphosphate concentration was not possible by our assay and, thus, it could not be determined to what extend the reaction follows a processive mechanism. Ishige et al. (2002) did not detect any intermediate chain lengths when utilizing polyphosphate with chain lengths of 750 phosphate units which suggests the reaction is highly processive. The same authors observed wide substrate acceptance with respect to polyphosphate chain lengths.

In our experiments, variation of enzyme and initial PolyP_14_ concentrations showed that their effect on the reaction rate is complex (see Figure 5a). This is in agreement with the reported enhanced enzyme activity by increased concentrations of polyphosphate and Mg^2+^ ions. Thus, it is not possible to model the kinetics over a wide range of PolyP_14_ and enzyme concentration by using a simple mechanistic model, for example mass action kinetics and Michaelis‐Menten‐type kinetics.

It was found that PolyP_14_ functions as an inhibitor above 6 mM (for His_6_‐1D‐Ppk2 0.05 mg/ml). Because of the limited solubility of the enzyme, final concentrations of 0.05 mg/ml (1.3 μM) were used. As can be seen from Figure 5b, at 4 mM PolyP_14_ and 0.05 mg/ml His_6_‐1D‐Ppk2, the phosphorylation reactions of ADP and GDP are independent of each other, for example, reaction rates are not affected by the presence of both nucleotides. Therefore, both reactions could be modeled independently and it was found that the experimental data with initial concentrations of 4 mM PolyP_14_ are best described by reversible mass action kinetics (see Table 1). In additional experiments, it was observed that at lower concentrations of PolyP_14_ (1–3 mM) the measured reaction rates were much lower than predicted by the kinetic equations. With 0.1 mM PolyP_14_, the substrate affinity (K_eq_) was higher for GDP than for ADP. This is in accordance with previous studies (Ishige et al., 2002; Nocek et al., 2008; Zhang et al., 2002). As 4 mM PolyP_14_ was found to be the optimal concentration, no attempt was made to establish a more complex rate equation to describe the inhibition of PolyP_14_ at initial concentrations above or below 4 mM.

No loss of enzyme activity was observed over 13 months when storing His_6_‐1D‐Ppk2 stock solutions at −20°C (data not shown).

#### 3.2.4 ManB‐His_6_/ManC

ManB catalyzes the conversion of man6P into man1P. ManC catalyzes the reaction of man1P and GTP into GDP‐man and pyrophosphate. *manB* and *manC* are neighboring genes in the polycistronic gene cluster for colanic acid biosynthesis in *Enterobacteriaceae* and both enzymes are usually used as a complex and produced in a bicistronic expression system (Koizumi et al., 2000; Lee et al., 2009). ManB and ManC from *Salmonella enterica* have been expressed in *E. coli*. Using purified enzymes, kinetic models for the reactions have been proposed and verified experimentally (Elling, Ritter, & Verseck, 1996; Fey, Elling, & Kragl, 1997). Because of the competitive inhibition of recombinant GDP‐man pyrophosphorylase of *S. enterica* for GTP by GDP‐man and the uncompetitive inhibition for man1P by GDP‐man (Yang et al., 2005), we investigated the production of recombinant ManB and ManC from *E. coli*. Since the protein sequences of ManB and ManC from *S. enterica* and *E. coli* differ significantly (ManB: 26.2% similarity/ 21.7% identity, ManC: 66.9% similarity/58.2% identity) kinetic parameters for the *E. coli* enzyme were determined (Table 1). Studies on ManB from *P. aeruginosa* found that the enzyme requires external glucose‐1,6‐bisphosphate and Mg^2+^ ions as cofactors for the transfer of phosphate groups between man1P and man6P (Naught & Tipton, 2001; Orvisky et al., 2003).

The equilibrium conversion of man1P to man6P catalyzed by the ManB‐His_6_/ManC complex was studied independently from the GDP‐man synthesis. It was found that ManB‐His_6_ does not require exogeneous glucose‐1,6‐bisphosphate but the reaction was faster with glucose‐1,6‐biphosphate. Moreover, glucose‐6‐phosphate peaks emerged when glucose‐1,6‐biphosphate was used. It is likely that there are multiple reaction equilibria between mannose‐phosphates and glucose‐phosphates as proposed in the literature for ManB from *Galdieria sulphuria* (Oesterhelt, Schnarrenberger, & Gross, 1997). In the presence of glucose‐1,6‐bisphosphate (0.25 mM) the reactions attained an equilibrium which is skewed toward man6P in less than 5 min for an initial concentration of 1.8 mM for man6P and man1P, respectively, and a ManB‐His_6_/ManC concentration of 0.035 mg/ml (0.7 µM for an estimated ManB/ManC ratio of 1:1).

Multiple experiments to elucidate the kinetics of the ManB‐His_6_/ManC complex were performed with various enzyme concentrations (0.086–0.855 mg/ml) and substrate concentrations (all between 0.4–1.7 mM). It was found that the reaction is reversible and that the equilibrium is shifted to the side of GTP and man1P when man1P is further converted to GDP‐man. Moreover, the rate of the GDP‐man synthesis by the ManB‐His_6_/ManC complex is governed by the conversion of pyrophosphate to phosphates. Reverse reactions were carried out for 1.7 mM GDP‐man and pyrophosphate, respectively, and for two different enzyme concentration (0.171 and 0.342 mg/ml). It was found that after rapid consumption of about 90% of the substrates, GDP‐man was again produced with a constant rate of 2.1 ± 0.4 μmol/min after 40 min due to cleavage of pyrophosphates (see Figure 4b).

The ManB‐His_6_/ManC enzyme complex did not lose activity within 7 months when stored at −20°C.

#### 3.2.5 Alg1ΔTM

Single‐enzyme reactions with 0.07–0.1 mg/ml (1.3–1.9 μM) Alg1ΔTM showed that 0.7 mM phytanyl‐PP‐(GlcNAc)_2_ and 1.5 mM GDP‐man are fully converted to phytanyl‐PP‐(GlcNAc)_2_‐Man_1_ and GDP within 20 min.

### 3.3 GDP‐mannose synthesis

#### 3.3.1 Simulation and experiment

Multi‐enzyme experiments were performed to demonstrate GDP‐man production by the cascade (see Figures 6 and 7). The model composed of the kinetics measured for the single‐enzyme reactions is compared against the experimental data (see Figure 6). While the fit between simulated and experimental data is reasonable for ADP and ATP, the model underestimates GDP‐man concentrations and overestimates GDP and GTP concentrations. One potential explanation is an increased activity of the ManB‐His_6_/ManC enzymes in the complex reaction mixture containing other enzymes and different concentrations of NaCl and imidazole. To confirm this explanation the mass action constant *k*_4_ was fitted to the experimental data of the cascade reaction while the other parameters were kept constant. The optimized *k*_4,opt_ was estimated to be 4.6 · 10^−3^ L^2^/(min g mol). The fact that this optimized model describes the cascade reactions accurately suggests an enhanced ManB‐His_6_/ManC activity in the multi‐enzyme reactions (see Figure 7a).

#### 3.3.2 Effect of pH value, temperature, and co‐factors on the one‐pot multi‐enzyme cascade reaction

To check the effect of pH value, temperature, and co‐factors on the GDP‐man synthesis, a number of one‐pot multi‐enzyme cascade reactions were carried out (see Figure 7). It was found that there is no significant difference in metabolite concentration profiles and, in particular, GDP‐man concentration over time at pH 7.0 (HEPES/NaOH), pH 7.5, and pH 8.0 (both Tris/HCl) (see Figure 7b). Increasing the temperature to 35°C did not change the rate of GDP‐man production within 240 min while at 25°C only about 200 μM (35% less) of GDP‐mannose was produced after 240 min (see Figure 7c). Moreover, it was found that ADP and GDP were not converted to ATP and GTP when the cascade reaction was initiated without addition of the co‐factor MgCl_2_ which indicates that His_6_‐1D‐Ppk2 requires MgCl_2_ for activity (data not shown). At 5 mM MgCl_2_ around 45% less GDP‐man was formed after 240 min compared to the reaction with 10 mM MgCl_2_ (see Figure 7d). Overall, the maximum amount of GDP‐man produced after 240 min was 566 μM (30°C, 10 mM MgCl_2_) with a constant reaction rate of 2.7 μM/min after a lag phase of 60 min. The initial concentrations were 0.8 mM GDP and 6 mM mannose and, thus, the yield with respect to GDP was 71%.

#### 3.3.3 Linking the GDP‐mannose cascade to Alg1 to produce phytanyl‐PP‐(GlcNAc)_2_‐Man_1_

**Figure 8:**
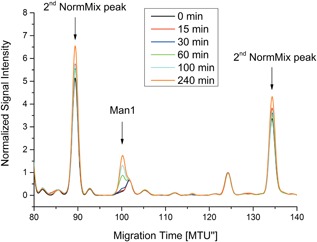
Electropherograms obtained by xCGE‐LIF of the multi‐enzyme cascade including Alg1ΔTM at different reaction time points. Reaction conditions as in Figure 7 plus 0.1 mg/ml Alg1ΔTM and 0.7 mM phytanyl‐PP‐(GlcNAc)_2_. The evolution of GlcNAc_2_‐Man_1_ (Man1) peaks at 101 MTU” is apparent. Migration times of 101 MTU” for GlcNAc_2_‐Man_1_ glycans are in agreement with the glyXtool™ database. Commercial LIZ™ base pair standard and 2nd NormMix from glyXera GmbH (Magdeburg, Germany) were used for the first and second migration time normalization. Peaks at 91 and 136 MTU” are 2nd NormMix peaks. The peak at 126 MTU” is an unknown impurity peak

The GDP‐man cascade was coupled with Alg1ΔTM to produce phytanyl‐PP‐(GlcNAc)_2_—Man_1_ starting from GDP, ADP, mannose, polyphosphate, and phytanyl‐PP‐(GlcNAc)_2_. xCGE‐LIF analyses of the samples shows an evolving Man1‐peak (GlcNAc)_2_‐Man_1_ over time at 101 MTU (see Figure 8) confirming the production of phytanyl‐PP‐(GlcNAc)_2_‐Man_1_. The migration time units were determined by mannosidase‐digested glycan standards (GlcNAc)_2_‐Man_4‐9_.

**Figure 9:**
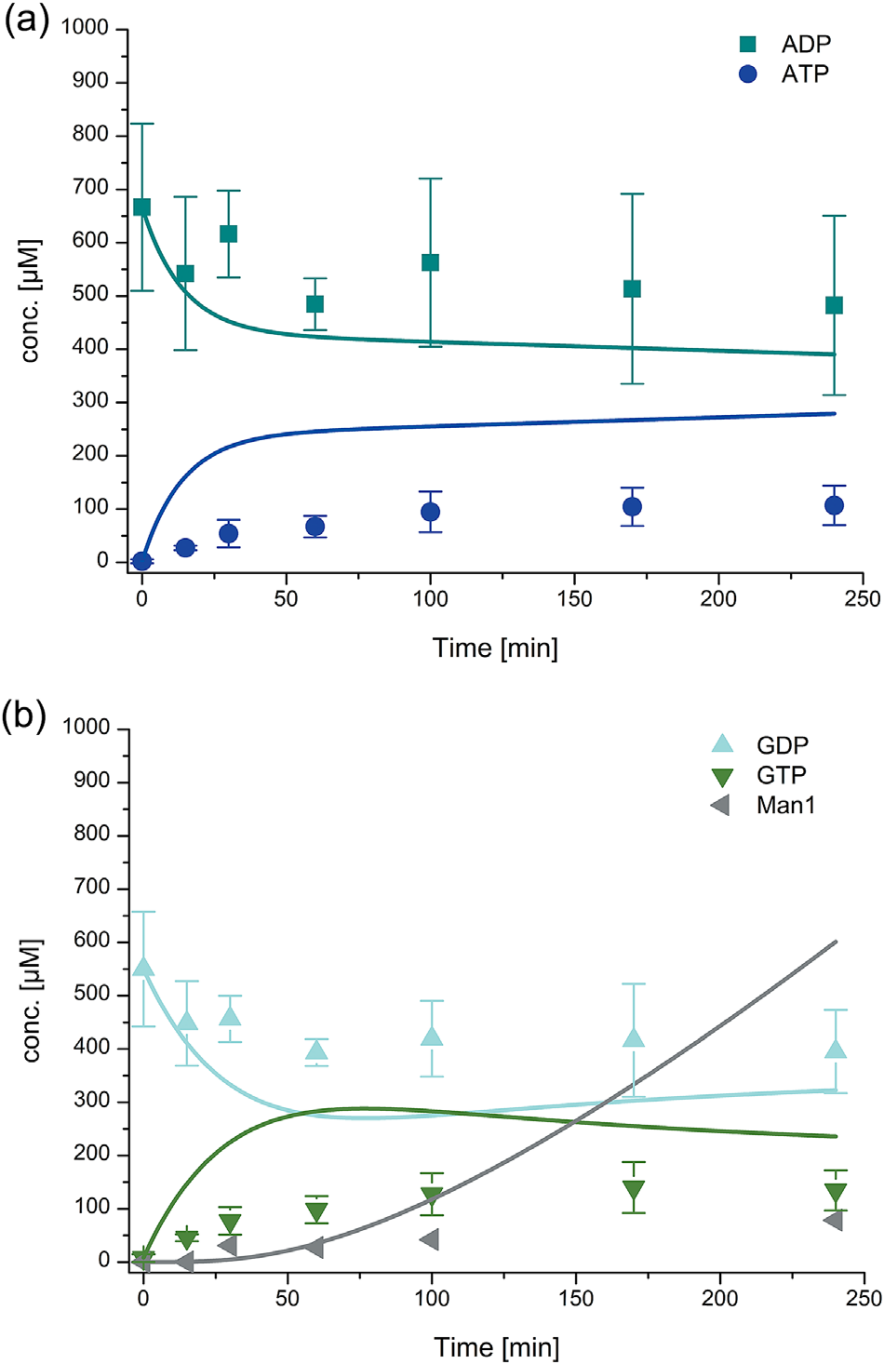
GDP‐mannose cascade coupled to Alg1ΔTM to produce phytanyl‐PP‐(GlcNAc)_2_‐Man_1_ (Man1) with continuous regeneration of GDP‐mannose. Experimental data (dots) and simulated data (lines). Reaction conditions as in Figure 7 plus 0.1 mg/ml Alg1ΔTM and 0.7 mM phytanyl‐PP‐(GlcNAc)_2_. Man1 concentrations are estimated from normalized relative peak areas of (GlcNAc)_2_ and Man1. The error bars represent the standard deviation of the mean calculated from three replicates. (a) ADP and ATP concentrations, (b) GDP, GTP, and Man1 concentrations

No GDP‐man was detected by HPAEC‐UV/CD which means that the GDP‐man to Man1 yield should be 100%. However, only about 80 μM phytanyl‐PP‐(GlcNAc)_2_‐Man_1_ was produced after 240 min which is equivalent to a yield of 11% with respect to phytanyl‐PP‐(GlcNAc)_2_. Thus, phytanyl‐PP‐(GlcNAc)_2_‐Man_1_ production in the multi‐enzyme cascade was much slower than predicted by the kinetic simulation (see Figure 9). Comparison between the simulation and the experimental data show that the steady‐state equilibrium of the His_6_‐1D‐Ppk2‐catalyzed conversions of ADP to ATP and GDP to GTP, respectively, is lower with respect to the nucleoside triphosphates than predicted. Parameter estimations reveal that the kinetic model fits the experimental data when constants *k*_2_ and *k*_3_ are adjusted to 0.097 and 0.041 L/(min g) values. This finding indicates that nucleoside diphosphate conversion is inhibited by phytanyl‐PP‐(GlcNAc)_2_, Alg1ΔTM or Triton X–100, which is present in the Alg1ΔTM stock solution. Triton X–100 is necessary to solubilize membrane‐bound enzymes and cannot be omitted.

## 4 CONCLUSIONS

A cell‐free cascade of five enzymes expressed in and purified from *E. coli* BL21‐Gold (DE3) was designed to produce and regenerate GDP‐mannose from mannose and polyphosphate with catalytic amounts of GDP and ADP. The maximum reaction rate of GDP‐mannose was 2.7 μM/min at 30°C and 10 mM MgCl_2_ producing 566 nmol GDP‐mannose from 800 nmol GDP and 6 μmol mannose after 240 min. Furthermore, it was demonstrated that the cascade can be in vitro coupled with a purified glycosyltransferase to donate mannose for the LLO assembly. A kinetic model based on single‐enzyme reactions was established to investigate inhibition and enhancement in the multi‐enzyme cascade. The model can be used to study and optimize coupling of the GDP‐mannose cascade with one or more glycosyltransferases. Overall, the study envisages a first step toward the development of a platform for the cell‐free production of LLOs as precursors for in vitro glycoengineering of proteins.

## Supporting information

Additional Supporting Information may be found online in the supporting information tab for this article.

**Figure S1**. SDS‐PAGE, Coomassie stained—elution fractions after IMAC purification of Alg1ΔTM.
**Figure S2**. Coverage of dimer peptides obtained from tryptic digestion of putative Alg1ΔTM dimer SDS‐PAGE band.
**Figure S3**. Coverage of peptides obtained from tryptic digestion of putative Alg1ΔTM monomer SDS‐PAGE band.
**Figure S4**. Conversion of pyrophosphate to phosphate by PmPpA. Initial concentrations of pyrophosphate are shown in the legend.
