# Supplementary material for: One pot synthesis of GDP‐mannose by a multi‐enzyme cascade for enzymatic assembly of lipid‐linked oligosaccharides
Source: Biotechnol Bioeng. 2017 Oct 23;115(1):192–205. doi: 10.1002/bit.26454 (PMC5765510; doi:10.1002/bit.26454)
Supplement: Supplementary file 1 — Figure S1. SDS‐PAGE, Coomassie stained—elution fractions after IMAC purification of Alg1ΔTM. Figure S2. Coverage of dimer peptides obtained from tryptic digestion of putative Alg1ΔTM dimer SDS‐PAGE band. Figure S3. Coverage of peptides obtained from tryptic digestion of putative Alg1ΔTM monomer SDS‐PAGE band. Figure S4. Conversion of pyrophosphate to phosphate by PmPpA. Initial concentrations of pyrophosphate are shown in the legend. [file BIT-115-192-s001.docx]

**Supplement**

**Chemicals**

The following materials were purchased from Sigma Aldrich (Munich, Germany): ADP (A2754, purity > 95%), ATP (A7699, > 99%), 8-aminopyrene-1,3,6-trisulfonic acid (ATPS) (09341, > 96%), GDP (G7127, > 96%), GDP-Man (G5131, > 97%), glucose-1-phosphat (G7000, > 97%), glucose-6-phosphat (G7250, > 98%), glucose-1,6-bisphosphat (glc1,6P) (49225, > 99%) GTP (G8877, > 95%), mannose-1-phosphate (man1P) (M1755, “Sigma grade”), and mannose-6-phosphate (man6P) (M6876, > 97%). All other chemicals were of analytical grade; base pair standard LIZ™ (4322679) and HiDi™-formamide (4311320) were purchased from Applied Biosystems (Waltham, USA); MgCl_2_ (A2264, > 99%), tris(hydroxymethyl)-aminomethan-buffer (TRIS) (A2264, > 99.9%), and Isopropyl β-D-1-thiogalactopyranoside (IPTG) (A1008,0025) were obtained from AppliChem (Darmstadt, Germany); HCl (4025, 37 %), Triton-X-100 (3051.4, “ultra pure”), and BSA (8076.2, ≥ 98%) from Carl Roth (Karlsruhe, Germany); Isopropanol (8.18766, > 99.5%) from Merck (Darmstadt, Germany); 2^nd^ NormMix from glyXera GmbH (Magdeburg, Germany); Sodium hexametaphosphate 68 (PolyP_14_) was a friendly gift from Thermphos International B.V. (Wittenberg, Germany); Phytanyl-PP-(GlcNAc)_2_ was chemically synthesized by ChiroBlock GmbH (Wolfen, Germany); Pierce™ Unstained Protein MW Marker used for SDS-PAGE (22610), GeneRuler 1kb DNA ladder (SM0311), GeneJET Plasmid Miniprep Kit (K0502), *Pfu* DNA Polymerase (EP0571), dNTP Mix (R0191), T4 DNA Ligase (EL0016), *NcoI* 500 Units (ER0571), *NdeI* 500 Units (ER0581), and *XhoI* 2.000 Units (ER0691) were purchased from Thermo Fisher Scientific (Dreieich, Germany).

**In-gel trypsin digestion**

The protein bands were cut out of the gels, incubated 45 min at 50°C with 10 mM dithiothreitol (Sigma, St Louis, USA) in 100 mM ammonium bicarbonate and after removing of the solution with 55 mM iodoacetamide from Sigma (St Louis, USA) in 100 mM ammonium bicarbonate for 45 min in the dark to modify cysteine residues. The solution was removed and the gel pieces washed three times with water, twice with 100 mM ammonium bicarbonate, and finally with 100 mM ammonium bicarbonate in 50 % acetonitrile. The gel pieces were dried, re-swollen in 20 µL 50 mM ammonium bicarbonate (pH 8.0) and digested with trypsin (Promega, Madison, USA) overnight at 37°C.

**Nano-HPLC**

Peptides were extracted from the gel pieces and injected into a nanoACQUITY UPLC system (Waters Co., Eschborn, Germany) equipped with a binary solvent manager, sample manager, heating and trapping module. 2 µL were injected via “microliter pickup” mode and desalted on-line through a Symmetry C18 180 µm × 20 mm pre-column. The peptides were separated on a 100 µm × 100 mm analytical RP column (1.7 µm BEH 130 C18, Waters Co., Milford, USA.) using a typical UPLC gradient from 3.0 % to 33.0 % over 15 min. The mobile phases used were 0.1 % formic acid in water and 0.1 % formic acid in acetonitrile. The column was connected to a SYNAPT® G2 MS (Waters Co., Milford, USA).

**ESI-QTOF-MS/MS-Analysis**

Data were acquired in LC/MS^E^ mode (an unbiased mobility-assisted TOF acquisition method) switching between low and elevated energy on alternate scans. Subsequent correlation of precursor and product ions can then be achieved using both retention and drift time alignment. Searches were conducted with GlobalSERVER™ against the SwissProt database and BiopharmaLynx (1.3.2, Waters Co., Milford, USA) was used to analyze the obtained data.

**Chromatography**

Injection volumes were 12.5 µL for all runs. Ion suppression to remove eluent and counter ions prior to detection was performed at 80 - 90 mA with electrolytically regenerated suppressor ERS™500.

KOH concentrations were generated by an EG40 eluent generator using an EGCII-KOH cartridge and degassing unit. All samples and external standards were injected three times for technical replication.

**Assay validation**

Assay validation was carried out according to Gottwald (Gottwald 2000). Six standard mixtures of all components were prepared in the range of 5 – 170 µM. Variances of the upper and lower concentrations were F-tested (P=99 %). It was found that variances were non-homogenous for all components (Draper and Smith 1981). Mandel’s fitting test (P=99 %) was used to confirm linearity of the regression (Sachs 2004). Standard deviation of the method was maximum 4.2 % for GTP.

**Gene and protein sequences**

*Nco*I-*ppa*-*Xho*I (coding for **PmPpA** from *Pasteurella multocida*, optimized for *E. coli*)

CCATGGGCCTGGAAACTGTTCCTGCTGGTAAAGCTCTGCCTGATGATATCTATGTAGTAATTGAAATCCCAGCAAACTCTGATCCAATTAAGTATGAAGTGGACAAAGAGTCTGGTGCGCTGTTCGTTGACCGCTTTATGGCTACTGCTATGTTCTACCCGGCGAACTACGGCTACGTTAACAACACTCTGTCTCTGGATGGCGACCCGGTGGATGTTCTGGTTCCGACTCCTTATCCGCTGCAGCCAGGTTCCGTAATCCGTTGTCGCCCTGTCGGCGTTCTGAAAATGACGGACGAAGCGGGTAGCGACGCGAAAGTAGTAGCAGTACCACACTCTAAACTGACCAAGGAATACGACCATATCAAGGACGTTAACGACCTGCCGGCTCTGCTGAAGGCCCAGATCCAGCACTTTTTCGAATCTTACAAGGCTCTGGAGGCGGGCAAATGGGTTAAAGTTGACGGCTGGGAGGGCGTTGATGCTGCGCGTCAGGAGATCCTGGATTCTTTCGAACGTGCAAAAAAGCTCGAG

Protein sequence of **PmPpA** from *Pasteurella multocida*

MGLETVPAGKALPDDIYVVIEIPANSDPIKYEVDKESGALFVDRFMATAMFYPANYGYVNNTLSLDGDPVDVLVPTPYPLQPGSVIRCRPVGVLKMTDEAGSDAKVVAVPHSKLTKEYDHIKDVNDLPALLKAQIQHFFESYKALEAGKWVKVDGWEGVDAARQEILDSFERAKKLE

*Nde*I-*ppk2*-*Sac*I (coding for **1D-Ppk2** from *Pseudomonas aeruginosa,* optimized for *E. coli*)

CATATGGATTCTTATGGTGATACTTCTGGTCGCATTGGTCGTGATTGGCTGGACCGCCACGACGAGGAACTGGAACAAGAACTGCTGGACGACGAACTGAACCTGGATGAACTGTTCGGCCCGGAACAAGAAGACGCTCCGGGTGAGCTGTCTCGCCGTCGCTACTTCCGTGAACTGTTCCGTCTGCAGCGTGAACTGGTTAAGCTGCAGAACTGGGTAGTCCACACTGGTCACAAAGTCGTCATTCTGTTCGAAGGTCGTGACGCGGCAGGTAAAGGTGGTGTGATCAAACGTATCACCCAGCGTCTGAACCCGCGCGTGTGCCGTGTAGCTGCTCTGCCAGCCCCGAACGACCGCGAGCAGACTCAGTGGTACTTCCAGCGTTACGTGTCCCATCTGCCGGCTGGTGGCGAAATTGTTCTGTTTGATCGTAGCTGGTACAACCGCGCAGGCGTGGAGCGTGTCATGGGTTTTTGCAACGACGAGCAGTACGAAGAATTCTTTCGTTCCGTACCAGAATTTGAGAAAATGCTGGCTCGTTCCGGTATTCAGCTGCTGAAGTATTGGTTCTCCATCTCCGACGCGGAACAGCATCTGCGTTTTCTGTCCCGCATCCACGACCCGCTGAAACAGTGGAAACTGTCCCCGATGGATCTGGAGTCTCGTCGCCGTTGGGAAGCGTATACGAAGGCAAAAGAAACGATGCTGGAACGTACTCACATTCCGGAGGCCCCATGGTGGGTGGTACAGGCAGACGATAAGAAACGTGCTCGTCTGAACTGCATCCATCACCTGCTGCAACAGATGCCGTATCGTGAAGTTCCGCAGCCGCCGGTACACCTGCCGGAACGCCTGCGTCACGCCGACTACGTGCGTCACCCGACCCCGGGTGAAATCATCGTTCCGGAAGTTTACTAAGAGCTC

Protein sequence of **1D-Ppk2** from *Pseudomonas aeruginosa*

MDSYGDTSGRIGRDWLDRHDEELEQELLDDELNLDELFGPEQEDAPGELSRRRYFRELFRLQRELVKLQNWVVHTGHKVVILFEGRDAAGKGGVIKRITQRLNPRVCRVAALPAPNDREQTQWYFQRYVSHLPAGGEIVLFDRSWYNRAGVERVMGFCNDEQYEEFFRSVPEFEKMLARSGIQLLKYWFSISDAEQHLRFLSRIHDPLKQWKLSPMDLESRRRWEAYTKAKETMLERTHIPEAPWWVVQADDKKRARLNCIHHLLQQMPYREVPQPPVHLPERLRHADYVRHPTPGEIIVPEVY

*Nde*I-*ALG1 (Δ4-105)* (coding for **Alg1ΔTM** from *Saccharomyces cerevisiae,* optimized for *E. coli*)

CATATGAAATCTACTAAGAAACGCATTATTATCTTTGTCCTGGGTGATGTTGGCCACAGCCCTCGTATTTGCTACCATGCAATTTCCTTCTCCAAACTGGGCTGGCAAGTGGAACTGTGCGGTTACGTGGAAGACACCCTGCCAAAAATCATCTCTTCCGATCCGAACATCACCGTTCATCACATGAGCAATCTGAAACGTAAAGGTGGCGGTACTTCCGTTATCTTCATGGTGAAGAAAGTGCTGTTCCAGGTTCTGTCTATCTTTAAGCTGCTGTGGGAACTGCGTGGTTCTGACTATATTCTGGTTCAGAACCCGCCGTCTATCCCGATTCTGCCGATCGCGGTCCTGTACAAACTGACTGGTTGCAAACTGATCATCGACTGGCATAACCTGGCTTACTCTATCCTGCAGCTGAAATTCAAAGGTAACTTTTACCACCCGCTGGTTCTGATCAGCTATATGGTTGAAATGATCTTTTCTAAATTCGCAGATTACAACCTGACTGTCACGGAAGCGATGCGCAAATATCTGATCCAGTCCTTCCATCTGAACCCGAAACGTTGTGCGGTGCTGTATGATCGTCCGGCTTCCCAGTTCCAACCGCTGGCAGGTGACATCAGCCGCCAGAAGGCCCTGACCACCAAAGCGTTTATCAAAAACTATATCCGTGATGATTTTGACACGGAAAAAGGTGATAAGATCATTGTGACTTCCACCAGCTTCACCCCGGACGAGGACATCGGCATTCTGCTGGGTGCACTGAAAATCTACGAAAACTCTTACGTTAAATTCGACTCCTCCCTGCCGAAAATTCTGTGCTTTATTACTGGCAAGGGCCCGCTGAAGGAAAAATACATGAAACAAGTAGAAGAATATGACTGGAAGCGTTGCCAGATTGAATTCGTCTGGCTGAGCGCAGAGGATTATCCGAAACTGCTGCAGCTGTGCGACTACGGTGTATCTCTGCATACCTCTAGCTCCGGCCTGGATCTGCCGATGAAAATCCTGGACATGTTCGGCAGCGGTCTGCCAGTCATCGCTATGAACTACCCTGTTCTGGATGAACTGGTCCAGCACAACGTTAACGGTCTGAAATTCGTCGATCGTCGTGAACTGCATGAATCCCTGATCTTCGCTATGAAAGATGCCGACCTGTACCAAAAACTGAAGAAAAACGTAACCCAAGAAGCAGAAAACCGCTGGCAAAGCAACTGGGAGCGCACCATGCGTGACCTGAAGCTGATCCACTAACTCGAG

Protein sequence of Alg1ΔTM from *Saccharomyces cerevisiae*

MKSTKKRIIIFVLGDVGHSPRICYHAISFSKLGWQVELCGYVEDTLPKIISSDPNITVHHMSNLKRKGGGTSVIFMVKKVLFQVLSIFKLLWELRGSDYILVQNPPSIPILPIAVLYKLTGCKLIIDWHNLAYSILQLKFKGNFYHPLVLISYMVEMIFSKFADYNLTVTEAMRKYLIQSFHLNPKRCAVLYDRPASQFQPLAGDISRQKALTTKAFIKNYIRDDFDTEKGDKIIVTSTSFTPDEDIGILLGALKIYENSYVKFDSSLPKILCFITGKGPLKEKYMKQVEEYDWKRCQIEFVWLSAEDYPKLLQLCDYGVSLHTSSSGLDLPMKILDMFGSGLPVIAMNYPVLDELVQHNVNGLKFVDRRELHESLIFAMKDADLYQKLKKNVTQEAENRWQSNWERTMRDLKLIH

Protein sequence of ManB from *E. coli* P24175-MANB_ECOLI Phosphomannomutase OS=Escherichia coli (strain K12)

MKKLTCFKAYDIRGKLGEELNEDIAWRIGRAYGEFLKPKTIVLGGDVRLTSETLKLALAKGLQDAGVDVLDIGMSGTEEIYFATFHLGVDGGIEVTASHNPMDYNGMKLVREGARPISGDTGLRDVQRLAEANDFPPVDETKRGRYQQINLRDAYVDHLFGYINVKNLTPLKLVINSGNGAAGPVVDAIEARFKALGAPVELIKVHNTPDGNFPNGIPNPLLPECRDDTRNAVIKHGADMGIAFDGDFDRCFLFDEKGQFIEGYYIVGLLAEAFLEKNPGAKIIHDPRLSWNTVDVVTAAGGTPVMSKTGHAFIKERMRKEDAIYGGEMSAHHYFRDFAYCDSGMIPWLLVAELVCLKDKTLGELVRDRMAAFPASGEINSKLAQPVEAINRVEQHFSREALAVDRTDGISMTFADWRFNLRTSNTEPVVRLNVESRGDVPLMEARTRTLLTLLNE

P26405-RFBK_SALTY Phosphomannomutase OS=Salmonella typhimurium (strain LT2 / SGSC1412 / ATCC 700720)

MNVVNNSRDVIYSSGIVFGTSGARGLVKDFTPQVCAAFTVSFVAVMQEHFSFDTVALAIDNRPSSYGMAQACAAALADKGVNCIFYGVVPTPALAFQSMSDNMPAIMVTGSHIPFERNGLKFYRPDGEITKHDEAAILSVEDTCSHLELKELIVSEMAAVNYISRYTSLFSTPFLKNKRIGIYEHSSAGRDLYKPLFIALGAEVVSLGRSDNFVPIDTEAVSKEDREKARSWAKEFDLDAIFSTDGDGDRPLIADEAGEWLRGDILGLLCSLALDAEAVAIPVSCNSIISSGRFFKHVKLTKIGSPYVIEAFNELSRSYSRIVGFEANGGFLLGSDICINEQNLHALPTRDAVLPAIMLLYKSRNTSISALVNELPTRYTHSDRLQGITTDKSQSLISMGRENLSNLLSYIGLENEGAISTDMTDGMRITLRDGCIVHLRASGNAPELRCYAEANLLNRAQDLVNTTLANIKKRCLL

Alignment P24175 ManB and P26405 RfbKp: Identity 21.7%, Similarity 26.2%

*E. coli* MK-----------KLTCFKAYDIRGKLGEELNEDIA----WRIGRAYGEFLKPKTIVLGG

*S. enterica* MNVVNNSRDVIYSSGIVFGTSGARGLVK-DFTPQVCAAFTVSFVAVMQEHFSFDTVALAI

*: . * : . ** : ::. ::. : . *.:. .*:.*.

*E. coli* DVRLTSETLKLALAKGLQDAGVDVLDIGMSGTEEIYFATFHLGVDGGIEVTASHNPMDYN

*S. enterica* DNRPSSYGMAQACAAALADKGVNCIFYGVVPTPALAFQSMSDN-MPAIMVTGSHIPFERN

* * :* : * * .* * **: : *: * : * :: . .* **.** *:: *

*E. coli* GMKLVREGARPISGDTG-LRDVQRLAEANDFPPVDETKRGRYQQINLRDAYVDHLFGYIN

*S. enterica* GLKFYRPDGEITKHDEAAILSVEDTCSHL---ELKELIVSEMAAVNYISRYTS-LFS---

*:*: * ... . * . : .*: .. :.* .. :* . *.. **.

*E. coli* VKNLTPLKLVINSGNGAAGPVVDAIEARFKALGAPVELIKVHNTPDGNFPNGIPNPLLPE

*S. enterica* TPFLKNKRIGIYEHSS-A--GRDLYKPLFIALGAEVVSLGR----SDNFVPIDTEAVSKE

. *. :: * . .. * * : * **** * : ..** : : *

*E. coli* CRDDTRNAVIKHGADMGIAFDGDFDRCFLFDEKGQFIEGYYIVGLLAEAFLEKNPGA---

*S. enterica* DREKARSWAKEFDLDAIFSTDGDGDRPLIADEAGEWLRGDIL-GLLCSLALDAEAVAIPV

*:.:*. . :.. * :: *** ** :: ** *:::.* : ***.. *: : *

*E. coli* ---KIIHDPRLSWNTVDVVTAAGGTPVMSKTGHAFIKERMRKEDAIYGGEMSAHHYFRDF

*S. enterica* SCNSIISSGRF-FKH----------VKLTKIGSPYVIEAFNELSRS---------YSRIV

.** . *: :: ::* * :: * :.: . * * .

*E. coli* AYCDSGMIPWLLVAELVCLKDKTLGELVRD------------------------RMAAFP

*S. enterica* GFEANG---GFLLGSDICINEQNLHALPTRDAVLPAIMLLYKSRNTSISALVNELPTRYT

.: .* :*:.. :*::::.* * : :

*E. coli* ASGEINS---KLAQPVEAINRVE--------QHFSREALAVDRTDGISMTFADW-RFNLR

*S. enterica* HSDRLQGITTDKSQSLISMGRENLSNLLSYIGLENEGAISTDMTDGMRITLRDGCIVHLR

*..::. . :* : ::.* : .. *::.* ***: :*: * .:**

*E. coli* TSNTEPVVRLNVESRG--------DVPLMEARTRTLLTLLNE

*S. enterica* ASGNAPELRCYAEANLLNRAQDLVNTTLANIKKRCLL-----

:*.. * :* .*:. :. * : :.* **

P24174-MANC_ECOLI Mannose-1-phosphate guanylyltransferase OS=*Escherichia coli* (strain K12)

MAQSKLYPVVMAGGSGSRLWPLSRVLYPKQFLCLKGDLTMLQTTICRLNGVECESPVVICNEQHRFIVAEQLRQLNKLTENIILEPAGRNTAPAIALAALAAKRHSPESDPLMLVLAADHVIADEDAFRAAVRNAMPYAEAGKLVTFGIVPDLPETGYGYIRRGEVSAGEQDMVAFEVAQFVEKPNLETAQAYVASGEYYWNSGMFLFRAGRYLEELKKYRPDILDACEKAMSAVDPDLNFIRVDEEAFLACPEESVDYAVMERTADAVVVPMDAGWSDVGSWSSLWEISAHTAEGNVCHGDVINHKTENSYVYAESGLVTTVGVKDLVVVQTKDAVLIADRNAVQDVKKVVEQIKADGRHEHRVHREVYRPWGKYDSIDAGDRYQVKRITVKPGEGLSVQMHHHRAEHWVVVAGTAKVTIDGDIKLLGENESIYIPLGATHCLENPGKIPLDLIEVRSGSYLEEDDVVRFADRYGRV

P26404-RFBM_SALTY Mannose-1-phosphate guanylyltransferase RfbM OS=*Salmonella typhimurium* (strain LT2 / SGSC1412 / ATCC 700720)

MSFLPVIMAGGTGSRLWPLSREYHPKQFLSVEGKLSMLQNTIKRLASLSTEEPVVICNDRHRFLVAEQLREIDKLANNIILEPVGRNTAPAIALAAFCALQNADNADPLLLVLAADHVIQDEIAFTKAVRHAEEYAANGKLVTFGIVPTHAETGYGYIRRGELIGNDAYAVAEFVEKPDIDTAGDYFKSGKYYWNSGMFLFRASSYLNELKYLSPEIYKACEKAVGHINPDLDFIRIDKEEFMSCPSDSIDYAVMEHTQHAVVIPMSAGWSDVGSWSSLWDISNKDHQRNVLKGDIFAHACNDNYIYSEDMFISAIGVSNLVIVQTTDALLVANKDTVQDVKKIVDYLKRNDRNEYKQHQEVFRPWGKYNVIDSGKNYLVRCITVKPGEKFVAQMHHHRAEHWIVLSGTARVTKGEQTYMVSENESTFIPPNTIHALENPGMTPLKLIEIQSGTYLGEDDIIRLEQRSGFSKEWTNERS

Alignment P24174 ManC and P26404 RfbMp: Identity 58.2%, Similarity 66.9%

*E. coli* MAQSKLYPVVMAGGSGSRLWPLSRVLYPKQFLCLKGDLTMLQTTICRLNGVECESPVVIC

*S. enterica* ---MSFLPVIMAGGTGSRLWPLSREYHPKQFLSVEGKLSMLQNTIKRLASLSTEEPVVIC

.: **:****:********* :*****.::*.*:***.** ** .:. *.*****

*E. coli* NEQHRFIVAEQLRQLNKLTENIILEPAGRNTAPAIALAALAAKRHSPESDPLMLVLAADH

*S. enterica* NDRHRFLVAEQLREIDKLANNIILEPVGRNTAPAIALAAFCALQNADNADPLLLVLAADH

*::***:******:::**::******.************:.* ::: ::***:*******

*E. coli* VIADEDAFRAAVRNAMPYAEAGKLVTFGIVPDLPETGYGYIRRGEVSAGEQDMVAFEVAQ

*S. enterica* VIQDEIAFTKAVRHAEEYAANGKLVTFGIVPTHAETGYGYIRRGELIGND----AYAVAE

** ** ** ***:* ** ********** ***********: ..: *: **:

*E. coli* FVEKPNLETAQAYVASGEYYWNSGMFLFRAGRYLEELKKYRPDILDACEKAMSAVDPDLN

*S. enterica* FVEKPDIDTAGDYFKSGKYYWNSGMFLFRASSYLNELKYLSPEIYKACEKAVGHINPDLD

*****:::** *. **:************. **:*** *:* .*****:. ::***:

*E. coli* FIRVDEEAFLACPEESVDYAVMERTADAVVVPMDAGWSDVGSWSSLWEISAHTAEGNVCH

*S. enterica* FIRIDKEEFMSCPSDSIDYAVMEHTQHAVVIPMSAGWSDVGSWSSLWDISNKDHQRNVLK

***:*:* *::**.:*:******:* .***:**.*************:** : : ** :

*E. coli* GDVINHKTENSYVYAESGLVTTVGVKDLVVVQTKDAVLIADRNAVQDVKKVVEQIKADGR

*S. enterica* GDIFAHACNDNYIYSEDMFISAIGVSNLVIVQTTDALLVANKDTVQDVKKIVDYLKRNDR

**:: * ::.*:*:*. :::::**.:**:***.**:*:*::::******:*: :* :.*

*E. coli* HEHRVHREVYRPWGKYDSIDAGDRYQVKRITVKPGEGLSVQMHHHRAEHWVVVAGTAKVT

*S. enterica* NEYKQHQEVFRPWGKYNVIDSGKNYLVRCITVKPGEKFVAQMHHHRAEHWIVLSGTARVT

:*:: *:**:******: **:*..* *: ******* : .**********:*::***:**

*E. coli* IDGDIKLLGENESIYIPLGATHCLENPGKIPLDLIEVRSGSYLEEDDVVRFADRYGRV--

*S. enterica* KGEQTYMVSENESTFIPPNTIHALENPGMTPLKLIEIQSGTYLGEDDIIRLEQRSGFSKE

. : ::.**** :** .: *.***** **.***::**:** ***::*: :* *

*E. coli* ------

*S. enterica* WTNERS

Alignment with Clustal Omega - Analysis Tool Web Services from the EMBL-EBI. (2013), Sequence Identity And Similarity – SIAS, Immunomedicine group, Madrid, Spain

**Alg1ΔTM sequence and the coverage of peptides after tryptic digestion of monomer and dimer bands**

Elution fractions of Alg1ΔTM purification were analyzed by Coomassie stained SDS-PAGE (Figure S1), red boxed bands were cut out, digested with trypsin and further analyzed by ESI-QTOF-MS/MS


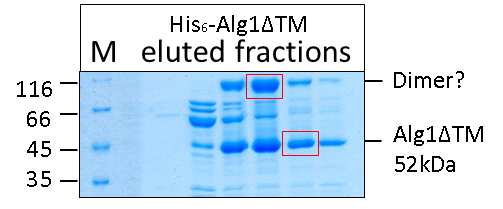


Figure S1: SDS-PAGE, Coomassie stained – elution fractions after IMAC purification of Alg1ΔTM


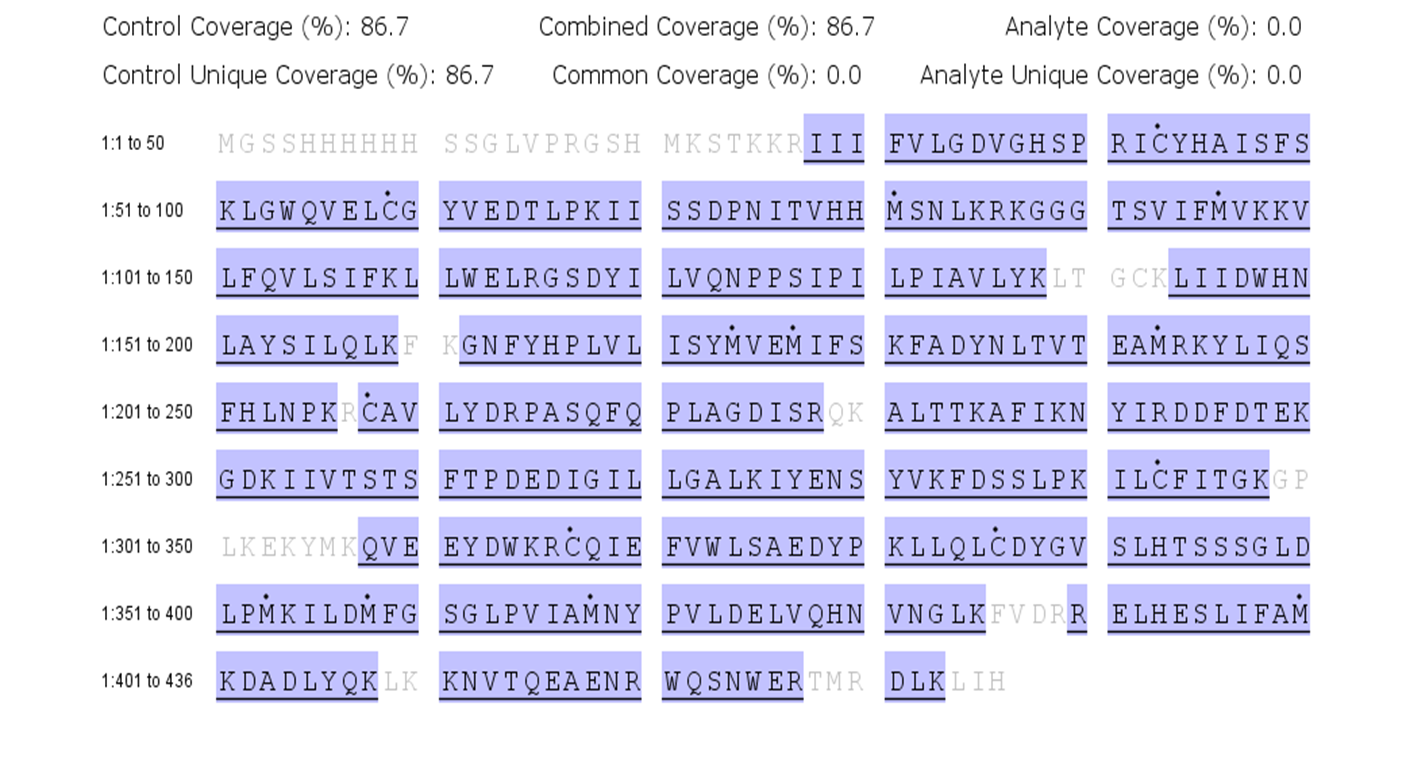


Figure S2: Coverage of dimer peptides obtained from tryptic digestion of putative Alg1ΔTM dimer SDS-PAGE band


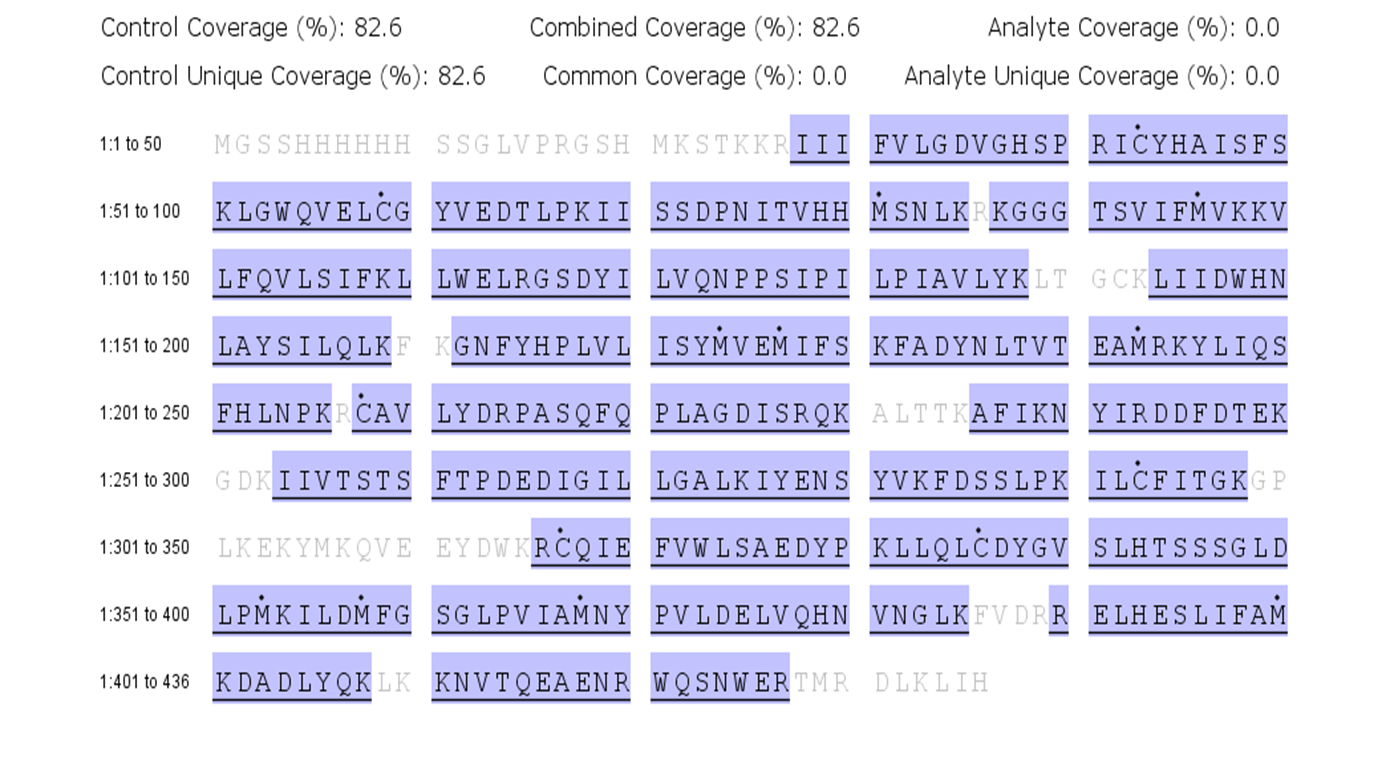


Figure S3: Coverage of peptides obtained from tryptic digestion of putative Alg1ΔTM monomer SDS-PAGE band

**PmPpA activity assay**

PmPpA activity was assayed by colorimetric measurement of ammonia molybdate complexed inorganic phosphate according to the literature (Taussky and Shorr 1953). The enzymatic reactions were carried out in a total assay volume of 1 mL containing 1.5 mM MgCl_2_, 35 mM TRIS/HCl at pH 7.5, various concentrations of pyrophosphate (0.150, 0.375, 0.750, 1.500, 2.250 μmol/mL) and 0.445 μg PmPpA. All substances were present in a volume of 900 μL. After preincubation at 35°C, the reactions were started by adding PmPpA in 100 μL TRIS buffer (50 mM, pH 7.5). Aliquoting and quenching was conducted by pipetting 100 μL of reaction mix into of 500 μL Taussky-Shorr-reagent* and 400 μL water. After incubation of this mixture for 2 minutes absorption was measured at 660 nm. The values were correlated against calibration curves of KH_2_PO_4_. The reaction time curves are shown in Figure S4. The time curves fitted Michaelis-Menten-type kinetics with substrate inhibition (see Equation S1). The maximum specific activity of PmPpa was 33.3 U/mg.

$$\frac{dp}{dt}=2 \frac{Vmax*[PP]}{K_{m}+\left[ PP \right]+\frac{{[PP]}^{2}}{K_{I}}}$$

Equation S1: Michaelis-Menten-type kinetics with substrate inhibition for the conversion of phosphate (P) to pyrophosphate (PP). Parameter are as follows: *V_max_* maximum reaction rate (14.8 µM/min), *K_m_* Michaelis-Menten constant (1.5 µM) and *K_I_* inhibition constant (89.1 µM).

* Taussky-Shorr-reagent:

1% Ammonium heptamolybdate tetrathydrate

0.5 M H_2_SO_4_

1.8 mM FeSO_4_


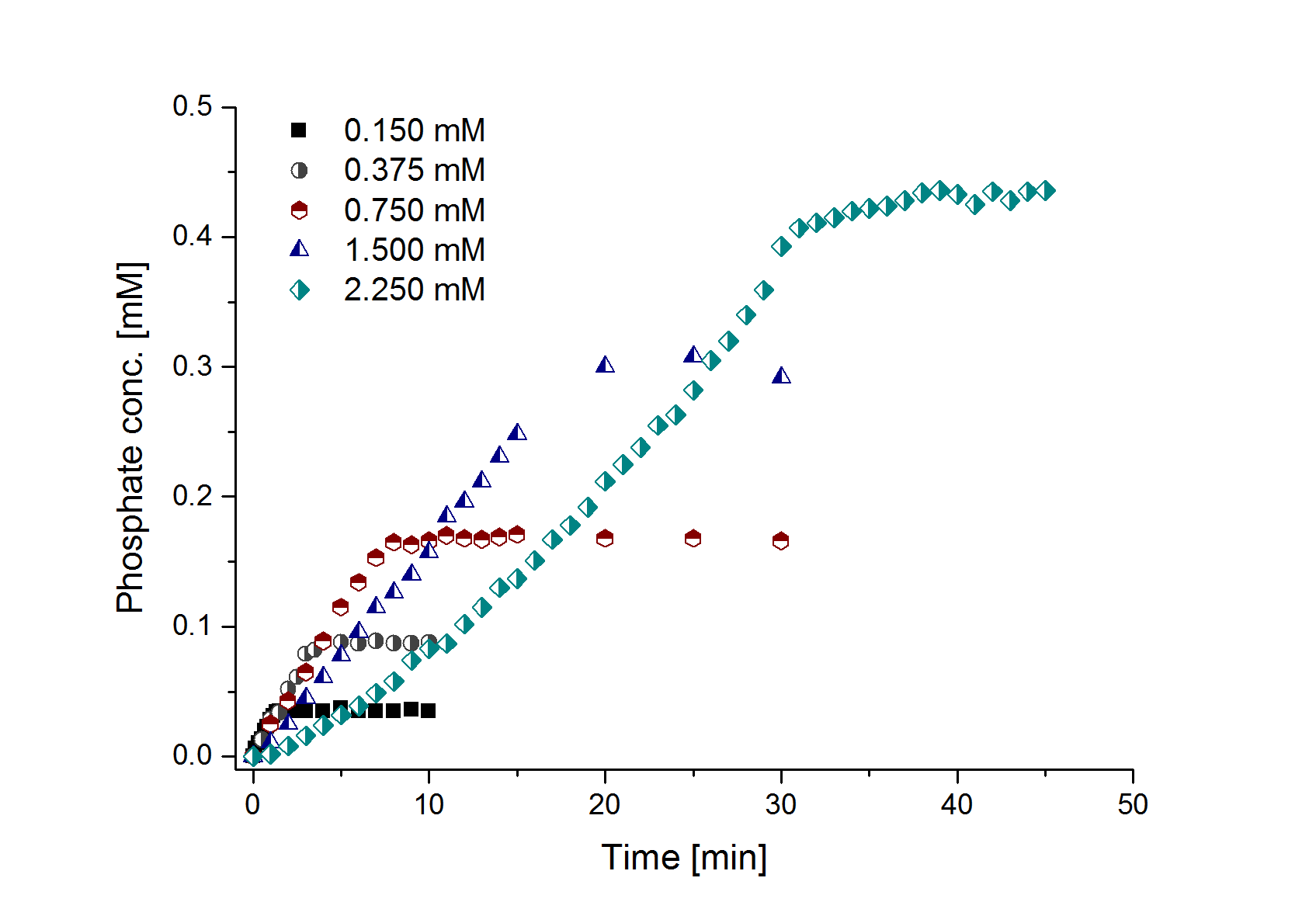


Figure S4: Conversion of pyrophosphate to phosphate by PmPpA. Initial concentrations of pyrophosphate are shown in the legend.

Draper NR, Smith H. 1981. Applied Regression Analysis: Wiley.

Gottwald W. 2000. Statistik für Anwender: Wiley-VCH.

Ritter JB, Genzel Y, Reichl U. 2006. High-performance anion-exchange chromatography using on-line electrolytic eluent generation for the determination of more than 25 intermediates from energy metabolism of mammalian cells in culture. Journal of Chromatography B 843(2):216-226.

Sachs L. 2004. Angewandte Statistik: Anwendung statistischer Methoden ; mit 317 Tabellen: Springer.

Taussky HH, Shorr E. 1953. A microcolorimetric method for the determination of inorganic phosphorus. J Biol chem 202(2):675-685.
